# Assessing Drug-Induced Mitochondrial Toxicity in Cardiomyocytes: Implications for Preclinical Cardiac Safety Evaluation

**DOI:** 10.3390/pharmaceutics14071313

**Published:** 2022-06-21

**Authors:** Xiaoli Tang, Zengwu Wang, Shengshou Hu, Bingying Zhou

**Affiliations:** 1 State Key Laboratory of Cardiovascular Diseases, Fuwai Hospital, National Center for Cardiovascular Diseases, Chinese Academy of Medical Sciences and Peking Union Medical College, 167 North Lishi Road, Xicheng District, Beijing 100037, China; tangxiaoli@fuwaihospital.org (X.T.); wangzengwu@foxmail.com (Z.W.); 2 Division of Prevention and Community Health, Fuwai Hospital, National Center for Cardiovascular Diseases, Chinese Academy of Medical Sciences and Peking Union Medical College, Mentougou District, Beijing 102308, China; 3 Department of Cardiovascular Surgery, State Key Laboratory of Cardiovascular Diseases, Fuwai Hospital, National Center for Cardiovascular Diseases, Chinese Academy of Medical Sciences and Peking Union Medical College, 167 North Lishi Road, Xicheng District, Beijing 100037, China

**Keywords:** mitochondria, drug toxicity, cardiomyocyte, high-throughput screening, preclinical cardiac safety assessment

## Abstract

Drug-induced cardiotoxicity not only leads to the attrition of drugs during development, but also contributes to the high morbidity and mortality rates of cardiovascular diseases. Comprehensive testing for proarrhythmic risks of drugs has been applied in preclinical cardiac safety assessment for over 15 years. However, other mechanisms of cardiac toxicity have not received such attention. Of them, mitochondrial impairment is a common form of cardiotoxicity and is known to account for over half of cardiovascular adverse-event-related black box warnings imposed by the U.S. Food and Drug Administration. Although it has been studied in great depth, mitochondrial toxicity assessment has not yet been incorporated into routine safety tests for cardiotoxicity at the preclinical stage. This review discusses the main characteristics of mitochondria in cardiomyocytes, drug-induced mitochondrial toxicities, and high-throughput screening strategies for cardiomyocytes, as well as their proposed integration into preclinical safety pharmacology. We emphasize the advantages of using adult human primary cardiomyocytes for the evaluation of mitochondrial morphology and function, and the need for a novel cardiac safety testing platform integrating mitochondrial toxicity and proarrhythmic risk assessments in cardiac safety evaluation.

## 1. Introduction

The heart, our central dispatcher of oxygen, nutrients, and drugs, is itself particularly susceptible to drug-induced toxicity. Cardiotoxicity is defined as the side effects of drugs that cause impairment of myocardial performance, which includes myocardial damage, abnormal electrical conduction, and secondary toxicity caused by drug effects on the vascular system or heart valves [1,2]. Cardiovascular toxicities due to therapeutic drugs comprise the group of toxicities with the highest incidence and severity among adverse drug reactions (ADRs) [3,4,5]. For example, 17% of drugs are halted at the preclinical stage due to cardiovascular toxicity [6]. In another report, drug discontinuation in non-clinical or clinical development related to cardiotoxicity reached 27–34% [1]. Despite increasing awareness of the variety of drug toxicities affecting the heart, their incidence in marketed drugs is 15–35% [1]. Notably, nearly 2000 marketed drugs have been associated with cardiovascular side effects, including ones with cardiovascular and noncardiovascular indications [7,8]. Sixty-nine drugs were withdrawn from the market due to serious cardiovascular ADRs between 1950 and December 2014 (Table 1).

The major clinical manifestations of cardiotoxicity are systolic or diastolic dysfunction and arrhythmia, the latter including abnormal cardiac rhythm disturbances in QT interval, bradycardia, and tachycardia [1,5,9,10]. The International Council for Harmonisation of Technical Requirements for Pharmaceuticals for Human Use (ICH) guidelines, including ICH S7B [9] and ICH E14 [10], were enacted to develop clinical and preclinical cardiotoxicity screening approaches in 2005, which significantly lowered the proportion of drugs with QT prolongation from 60% in 2005 to 10% in 2012 [11]. However, another 17 cardiotoxic drugs were withdrawn from the market following their implementation, including benfluorex (2009), rosiglitazone (2011), celecoxib (2011), ponatinib (2013), and etoricoxib, which have been reported to cause mitochondria dysfunction [12,13,14,15,16]. Thus far, 29% of withdrawn drugs have been reported to exhibit mitochondrial toxicity (Table 1). All current guidelines for standardizing the detection of cardiotoxicity, however, are still directed at arrhythmic risks.

Mitochondria are the metabolic centers of cells, performing fatty acid oxidation, the tricarboxylic acid (TCA) cycle, oxidative phosphorylation (OXPHOS) for ATP synthesis, heme biosynthesis, and amino acid metabolism. In addition, they also play an important role in the regulation of intracellular homeostasis, such as calcium homeostasis, biologic redox equilibrium, hormonal signaling, and apoptosis [17]. Cardiomyocyte, harboring up to 6000 mitochondria [18] that occupy 30–40% of the cell volume, is one of the highest ATP-consuming cell types. Much of the generated energy is used to sustain contraction [19] to supply blood through the circulatory system [20]. It is this high reliance on energy production that render cardiomyocytes particularly vulnerable to mitochondrial toxicants [21]. Mitochondrial toxicants are compounds that interrupt normal mitochondrial functions, resulting in compromised mitochondrial homeostasis, including disruption of oxidative phosphorylation, permeability transition, and generation of mitochondrial oxidative stress, contributing to energy supply disorder, aberrant intracellular signaling, toxic substances accumulation, autophagy or mitophagy disturbances, and programmed cell death, ultimately decreasing cardiac function [22,23,24,25]. At the organ level, many cardiac abnormalities are induced via these mechanisms, including cardiomyopathy [26,27,28], coronary heart disease [29,30], arrhythmias [31,32], ischemia reperfusion [33,34], and heart failure [35,36]. Mitochondrial impairment can adversely impact cardiomyocyte electrical excitability through mitochondrial gene expression alteration [37], mitochondrial membrane potential (MMP) collapse [38], excessive ROS generation [39], and ATP depletion [40], resulting in cardiac arrhythmias [41,42]. Therefore, mitochondria play important roles in the pathogenesis and development of various heart diseases and are common targets in promoting cardiotoxicity in both animal and cell culture [22,43,44,45]. Understanding and monitoring drug-induced mitochondrial cardiotoxicity constitutes a major part of cardiac safety and is critical to modern drug development. It is possible that a standardized approach for assessing non-arrhythmic toxicities, such as mitochondrial toxicity, might mitigate the occurrence of unexpected cardiotoxicity. 

In this review, we introduce the many aspects of mitochondrial biology and representative drugs that have been associated with cardiac toxicity. We elaborate on currently used detection methods of mitochondrial toxicity and discuss how these assays could be multiplexed in a high-throughput manner in cardiomyocytes. Finally, we propose ways to enhance our ability to identify mitochondrial liabilities of drugs as early as possible in the drug discovery process.

**Table 1:** List of drugs withdrawn from the market due to cardiovascular toxicity and their association with mitochondrial toxicity.

Medicinal Product	Class	Mechanism of Action	Launch Date	Year Withdrawn	Side Effects on Cardiac Function	Mitohondrial Toxicity
Amfepramone	Psychostimulant	Norepinephrine-releasing agent	1957	1975	-	Unknown
Benfluorex	Psychostimulant, anorectic, and hypolipidemic	Blocking of 5-HT2B	1976	2009	Valvular heart disease	Decrease in CPT I expression [46]
Emetine (ipecac syrup)	Emetic	Stimulation of the CTZ, local irritation	1912	1982	-	Unknown
Mephenesin	Muscle relaxant	Spinal reflex inhibition	1948	1976	-	Unknown
Rofecoxib	NSAID	COX-2 inhibitor	1999	2004	MI, cardiovascular thrombotic events, sudden death	Unknown
Adenosine phosphate	Antiarrhythmic	Direct nodal inhibition	1930	1973	-	Unknown
Alphacetylmethadol	Analgesic	OP1 receptor agonist	1993	2003	-	Unknown
Bepridil (Bepridil Hydrochlonde)	Antiarrhythmic, antianginal	Calcium channel blockers	1981	2004	Prolonged QT, TdP	Unknown
Budipine	Antiparkinsonian	Muscarinic and NMDA receptor antagonist	1979	2000	-	Unknown
Cliobutinol	Antitussive	Unclear	1961	2007	-	Unknown
Dofetilide	Antiarrhythmic	Inhibition of KCNH2, KCNK2, KCNJ12	1999	2004	QT prolongation, TdP	Unknown
Dolansetron	Propulsive	5-HT3 receptor antagonist	1997	2011	-	Unknown
Encainide	Antiarrhythmic	Na channel blocker	1985	1991	QT prolongation, TdP	Unknown
Grepafloxacin (Grepafloxacin Hydrochloride)	Antimicrobial	Inhibition of DNA gyrase	1997	1999	QT prolongation	Unknown
Indoramin	Vasodilator	Alpha-1 adrenoceptor antagonist	1981	2011	-	Unknown
Isoprenaline	Cardiac stimulant	Non-selective beta-adrenergic agonist	1949	1992	-	mPTP opening [47]
						Inhibition of OXPHOS [48]
Levacetylmethadol	Antidote	Mu-opioid receptor agonist, nicotinic acetylcholine receptor antagonist	1995	2001	-	Unknown
Nifedipine (10 mg)	Antihypertensive, antiemetic	Calcium channel blockers	1975	1996	Hypertension, angina, MI, CHF	Inhibition of ATP synthase [48]
Orciprenaline (metaprotenerol)	Bronchodilator	β2 adrenoceptor agonist	1961	2009	Tachycardia, palpitations	Unknown
Pergolide Mesylate	Anti-parkinsonian	Dopamine receptor agonist	2002	2007	Valvular heart disease	Unknown
Rosiglitazone	Hypoglycemic	Gluconeogenesis decrease	1999	2011	CHF, MI	Inhibition of ETC [48]
						Increase in mitochondrial oxidative stress, impairment of mitochondrial bioenergetics [13]
						Inhibition of complex I; uncoupling of OXPHOS [13]
Sibutramine (Sibutramine Hydrochlonde Hydrate)	Psychostimulant	Serotonin-norepinephrine reuptake inhibitor	2001	2002	Myocardial infarction	Increase in ROS formation [49]
Technetium (99mTc) fanolesomab	Radiography	Radioisotope	2004	2005	Cardiopulmonary arrest	Unknown
Tegaserod (Tegaserod Maleate)	Antispasmodic	5-HT4 receptor agonist	2002	2007	HF, ischemia	Unknown
Terodiline	Antispasmodic	Calcium channel blockade, blocks cholinergic receptor	1965	1991	Ventricular tachycardia, cardiac death	Unknown
Sertindole	Antipsychotic	5HT and D2 receptor antagonist/blocking of DRD2,HTR2A, HTR2C, HTR6	1996	1998	QT prolongation, TdP, sudden cardiac death	Unknown
Cloforex	Psychostimulant	Similar to amphetamine	1965	1967	-	Unknown
Astemizole	Antihistamine	H1-receptor antagonist, inhibition of KCNH2	1977	1987	long QT syndrome, TdP	Unknown
Cisapride monohydrate	Prokinetic agent	5-HT4 receptor agonist; inhibition of KCNH2	1993	2000	Ventricular arrhythmia, QT prolongation, TdP, cardiac arrest	Unknown
Tranylcypromine	Antidepressant	MAOI	1961	1964	-	Unknown
Bromocriptine mesylate	Anti-lactation	D2 and D3 agonist	1976	1989	-	Swollen mitochondria [50]
Domperidone (injectable)	Propulsive	Dopamine receptor antagonist	1979	1985	-	Unknown
Mepazine	Antiepileptic	Unclear	1955	1970	-	Unknown
Clozapine	Antipsychotic	Blocking of DRD2, HTR2A, DRD1, DRD3, DRD4, HTR1A, HTR1B, HTR1D, HTR1E, HTR2C, HTR3A, HTR6, HTR7, HRH1, HRH4, ADRA1A, ADRA1B, ADRA2A, ADRA2B, ADRA2C, CHRM1, CHRM2, CHRM3, CHRM4, CHRM5	1972	1975	Cardiomyopathy, MI, myocarditis, arrhythmia, Prolonged QT, TdP, cardiomyopathy	Inhibition of the ETC [51]
						Increase in ROS formation, GSH depletion, mitochondrial dysfunction, and swelling [52]
Vincamine	Nootropic	Unclear	1955	1980	-	Unknown
Lysine amidotriazoate	Radiography	-	1975	1995	-	Unknown
Terfenadine	Antihistamine	H1-receptor antagonist	1985	1997	QT prolongation, TdP	Increase in mtROS formation [53]
						MMP collapse [54]
Naftidrofuryl oxalate (IV)	Vasodilator	5HT2 receptor antagonist	1974	1992	-	Unknown
Cobalt	Hematinic	As cobalamin	1951	1967	-	Interruption of TCA and interference with the MRC enzymes [54]
						MMP collapse [55]
Chloroform (trichloromethane)	Anesthetic	Depression of the respiratory centres	1847	1976	-	MMP collapse [56]
						Megamitochondria [57]
Dithiazanine iodide	Antihelminth	Interruption of glucose uptake in cells	1959	1964	Prolonged QT, TdP	Unknown
Epinephrine (topical)	Anesthetic	Vasoconstriction	1899	2004	-	Unknown
Methylhexanamine (DMAA)	Nasal decongestant	Norepinephrine and dopamine transporter blockade	1948	1983	-	Unknown
Dexfenfluramine	Psychostimulant	Serotonin receptor agonist	1995	1997	Valvular heart disease, cardiac fibrosis	Unknown
Fenfluramine	Psychostimulant	Serotonin receptor antagonist	1973	1997	valvular heart disease	Mitochondrial fragmentation [58]
Parecoxib	Analgesic	COX-2 inhibitor	2002	2005	-	-
Prenylamine	Antianginal	Calcium channel blocker	1960	1989	QT prolongation, sudden cardiac death, ventricular tachycardia, TdP	Inhibition of FAO [59]
Probucol	Antioxidant	Inductor of LDL catabolism	1980	1989	QT prolongation, arrhythmias	Unknown
Droperidol	Antipsychotic	Dopamine 2 receptor antagonist	1970	2001	-	Unknown
Valdecoxib	NSAID	COX-2 inhibitor	2001	2005	Cardiomyopathy, CHF, hypertension, angina, arrhythmia	Inhibition of OXPHOS, mPTP opening [16]
Celecoxib (Onsenal)	NSAID	COX-2 inhibitor	2003	2011	-	Decrease in mitochondrial complex IV activity and induces oxidative stress [14]
						Increase in ROS formation, MMP collapse, mitochondrial swelling, ATP depletion [60]
						Suppression of mitochondrial function [61]
Bismuth salts	Antidyspepsia	Unclear. Forms insoluble complexes	1875	1978	-	Unknown
Levarterenol	Vasopressor	L-norepinephrine analogue	1904	1973	-	Unknown
Pipradrol	Psychostimulant	Norepinephrine-dopamine reuptake inhibitor	1953	1982	-	Unknown
Pseudoephedrine	Sympathomimetic	Direct action on adrenergic receptors	1959	2008	-	Unknown
Gallopamil	Antiarrhythmic	Calcium channel blockers	1983	2001	-	Decrease in mitochondrial biogenesis and mass [62]
Chlorphentermine	Psychostimulant	TAAR1 agonist, blocking of 5-HTs	1962	1969	Pulmonary heart disease	Inhibition of OXPHOS, uncoupling of OXPHOS [63]
Thioridazine	Antipsychotic	5HT2 receptor antagonist	1959	2000	QT prolongation, TdP, sudden cardiac death	mPTP opening [64]
						MMP collapse [65]
Buflomedil	Vasodilator	A-adrenergic blockade	1970	2006	QT prolongation, cardiac arrest	Unknown
Ponatinib Hydrochloride	Antineoplastic	Multi-target kinase inhibitor	2012	2013	-	Impairment of respiratory chain, increase in ROS formation, MMP collapse, mitochondrial fission [66]
Levomethadyl acetate	Analgesic (central nervous system agents)	Activation of OPRM1	1993	2002	QT prolongation, TdP	Unknown
Mesoridazine Besylate	Antipsychotic		1970	-	-	Unknown
Clobutinol Hydrochloride	Antitussive	Inhibition of GABA receptors	1961	2007	QT prolongation	Unknown
Phentermine	Central nervous system agents	Inhibition of SLC6A2, SLC6A3, SLC6A4; blockingof MAOA, MAOB	1959	1997	Valvular heart disease	Unknown
Mibefradil	Antihypertensive	Calcium channel blockers	1997	1998	QT prolongation	Unknown
Sparfloxacin	Antibiotics	Inhibits DNA gyrase	1997	2001	QT prolongation	MMP collapse [67]
Etoricoxib	Anti-inflammatory agents	Inhibition of COX-2	2002	2007	thrombotic events	Inhibition of OXPHOS [16]
Propoxyphene	Central nervous system agents	Activation of OP1, OP2, OP3	1957	2010	QT prolongation, TdP	Unknown
Lidoflazine	Cardiovascular agents	Blocking of calcium channels	1973	1989	QT prolongation	Unknown

## 2. Main Properties of Mitochondria and Drug-Induced Mitochondrial Toxicity in Cardiomyocytes

### 2.1. Morphology, Classification, and Structural Features of Mitochondria

**Figure 1:**
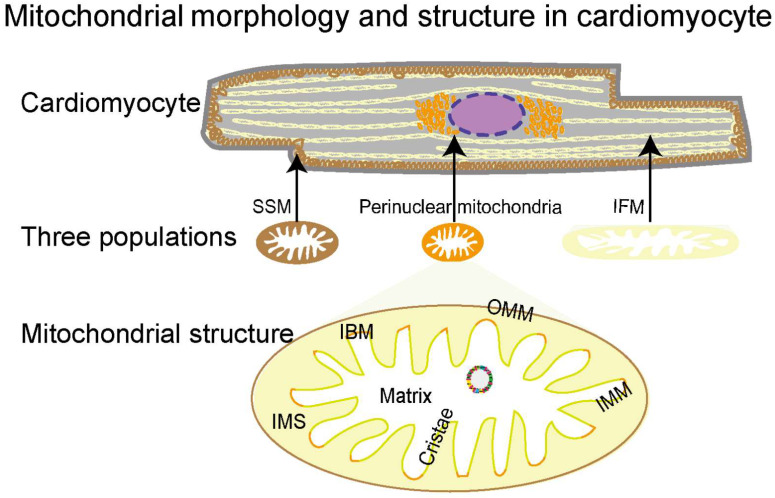
Mitochondrial morphology and structure in cardiomyocytes. Mitochondria in cardiomyocytes can be categorized into three subtypes, subsarcolemmal mitochondria (SSMs) (in brown), interfibrillar mitochondria (IFMs) (in yellow), and perinuclear mitochondria (in orange), according to their distribution, size, and shape. The mitochondrial double-membrane consists of an outer mitochondrial membrane (OMM) (brown) and an inner mitochondrial membrane (IMM) (green and orange). The space between the OMM and the IMM is the IMS, and inside the IMM is the matrix. The IMM consists of the inner boundary membrane (orange) and cristae (green), the latter of which are formed by extensive inward folding of the IMM.

The densely packed mitochondria in the cardiomyocyte provide over 95% of the energy for the heart to pump blood around the body [68,69]. They are highly dynamic organelles that quickly adjust their morphology, protein expression, and activity in response to the cellular environment [70,71,72]. Based on intracellular localizations, mitochondria in adult cardiomyocytes are classified into three populations: perinuclear, subsarcolemmal mitochondria (SSMs), and interfibrillar mitochondria (IFMs) (Figure 1) [68]. With distinctive locations, the three populations present unique morphologies and functions for the nucleus, cellular functions, and myofibril contraction, respectively [73,74,75,76]. Mitochondria are double-membrane organelles, consisting of structurally and functionally different membranes, that is, the outer mitochondrial membrane (OMM) and the inner mitochondrial membrane (IMM) (Figure 1). The OMM and IMM establish five compartments to provide unique biochemical environments for different functions, including the OMM, intermembrane space (IMS), IMM, cristae, and matrix [77,78]. The OMM forms a unique biochemical environment for diverse functions including coordination of protein import, mitochondrial dynamics, and inter-organellar communication. The IMS, the small volume between OMM and IMM, facilitates the translocation, folding, and post-translational events of nuclear DNA-encoding proteins into the matrix. Unlike the OMM permeabilizing molecules of up to 5 kDa [79], the IMM, a highly selective membrane, tightly controls the exchange of ions and metabolites through specialized molecular machinery. The IMM is essential for electron transport since it is necessary for the development of the proton motive force used for ATP generation. The cristae are formed by extensive inward folding of the IMM, increasing the membrane surface 1.5–2 fold to accommodate multi-enzyme complexes for ATP generation, especially the five bioenergetic complexes constituting the OXPHOS system [80]. The mitochondrial matrix is a complex mixture harboring several metabolic processes, including the tricarboxylic acid cycle (TCA), mitochondrial fatty acid oxidation (mtFAO), OXPHOS, Fe-s cluster biogenesis, heme synthesis, and many others. In addition, the matrix also hosts the mitochondrial genome (mtDNA), RNA, and ribosomes. Together, these five compartments coordinate with each other to perform mitochondrial functions, and their structural integrity is essential for healthy mitochondria.

Ultrastructural analysis by transmission electron microscopy (TEM) is a traditional method for the assessment of mitochondrial architecture. In addition, MitoTracker or fluorescently labeled mitochondrial antibodies have been applied to automated high-content imaging of mitochondria, and the resulting mitochondrial scores correlated well with cytotoxicity [81]. These methods are efficient at detecting several structural abnormalities induced by mitochondrial toxicants, including both oncological and non-oncological drugs. Of the non-oncological drugs, isoproterenol induces mitochondrial swelling, cristae disappearance, and matrix cavitation in cardiomyocytes [82,83]. Mitochondrial swelling and mitochondrial membrane rupture occurred in cardiomyocytes of As_2_O_3_-treated mice [84]. Diclofenac [85] and remdesivir [86] treatment also result in mitochondrial damages, as indicated by MitoTracker. Based on immunofluorescence analysis of TOM20, mitochondrial distribution and mitochondrial network disruption, as well as mitophagy, were revealed in nicotine-treated neonatal rat ventricular myocytes [87,88]. Oncological drugs have been frequently reported to cause structural damage to mitochondria. TEM revealed mitochondrial swelling, electron-dense matrix deposits, and matrix clearout in rats given sunitinib, and disrupted mitochondrial cristae in rats given sorafenib [89]. Regorafenib was also reported to induce electron-dense mitochondria and matrix cavitation [90]. MitoTracker indicated mitochondrial damage in cardiomyocytes induced by imatinib [91] and sunitinib [92]. The high sensitivity of mitochondrial structure to functional aberrations makes structural abnormality one of the most commonly observed manifestations of mitotoxicity.

The use of DOX, a commonly used chemotherapeutic anti-cancer drug of the anthracycline family, has been greatly limited because one-fourth of patients have experienced DOX-induced cardiotoxicity, including CHF, decreased LVEF, sinus tachycardia, myocarditis, and cardiomyopathy [93]. Numerous animal- and cardiomyocyte-based studies have revealed DOX-induced abnormal mitochondrial morphology and structure. Abnormal mitochondrial morphology, including mitochondrial swelling, cristae disappearance, and matrix cavitation, was found in doxorubicin (DOX) [94,95,96]. Microscopic evaluation by MitoTracker staining of mitochondria pinpointed the distribution of daunorubicin and DOX [97,98]. Based on immunofluorescence analysis of TOM20, mitochondrial distribution and mitochondrial network disruption, as well as mitophagy, were revealed in DOX-treated neonatal rat ventricular myocytes [87,88]. To mimic human anticancer therapy, the administration schedule was conducted by multiple administrations at separate time points, including 3 mg/kg every other day for a cumulative dose of 9 mg/kg [99], twice a week for three weeks to attain a 9 mg/kg cumulative dose [100], 6 doses of 3 mg/kg [101], 2–2.5 mg/kg/48 h over 12 days [94], 2 mg/kg for 10 consecutive days [102], 5 mg/kg once a week to a total of 20 mg/kg [103], or two doses of 10 mg/kg [104]. Of note, a cumulative dose of 20 mg/kg in adult mice roughly corresponds to 120 mg/m^2^ in humans, which is much lower than the maximum lifelong dose of 400–550 mg/m^2^ [105]. Yet even at these low dosages, cardiomyocytes had swollen mitochondria, loss of mitochondrial membrane integrity and cristae, cristae disarrangement, and/or clear matrix, suggesting the strong mitochondrial toxicity of DOX. DOX has been reported to cause acute cardiotoxicity when administered at a dose of 15 mg/kg or greater [106]. Mitochondria showed vacuolization, or even complete loss of the cristae, 48 h after a single dose of 20 mg/kg DOX injection into rats [107]. Fourteen days’ administration of DOX (20 mg/kg, single dose) in C57BL/6 mice resulted in mitochondrial oedema [108]. To investigate chronic cardiotoxicity, five doses (3 mg/kg each, bi-weekly) of DOX were given to C57BL/6 mice. Three months after the first dose, the authors observed hyperproliferation of mitochondria in cardiomyocytes [109]. It is evident from these studies that, even with different treatment schedules and dosages, mitochondrial structure is a stable indication for mitotoxicant assessment. Additional details, including maximum serum concentration (C_max_), cardiotoxicity manifestations, experimental models, and so on, of drugs affecting mitochondrial morphology and structure are listed in Table 2.

### 2.2. Substrate Catabolism and OXPHOS

**Figure 2:**
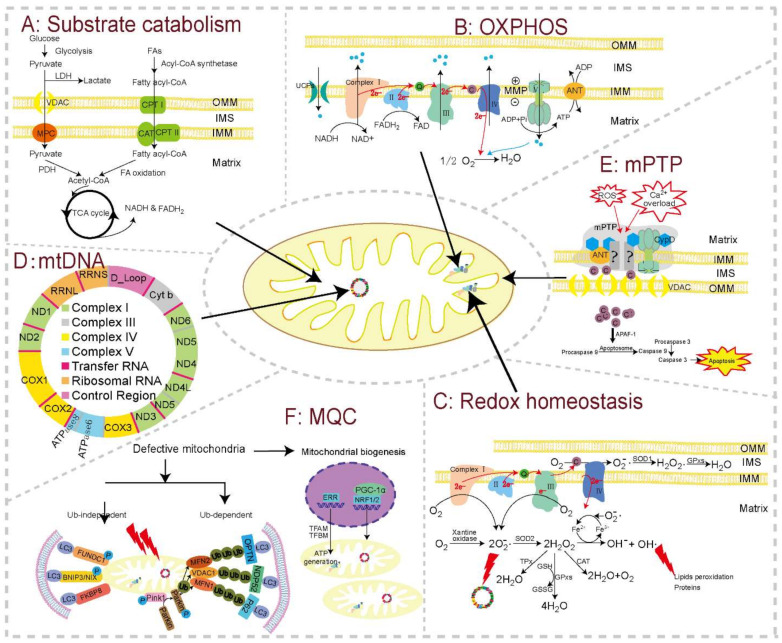
The basic modules of mitochondrial function and major toxicity targets in cardiomyocytes. Substrate catabolism, oxidative phosphorylation (OXPHOS), redox homeostasis, mitochondrial genome (mtDNA), mitochondrial permeability transition pore (mPTP), and mitochondrial quality control (MQC) constitute the major functional units and toxicity targets in cardiomyocytes. (**A**) Substrate catabolism. Fatty acids, esterificated by fatty acyl-CoA synthase enzymes, are taken up through CPT I, CPT II, and CAT, and are then oxidized into acetyl-CoA inside the mitochondrion. Pyruvate from glycolysis is also oxidized into acetyl-CoA by PDH in the mitochondrion. Acetyl-CoA then goes through TCA cycle to generate reducing equivalents (NADH and FADH_2_), which fuel OXPHOS to produce ATP. This bioenergetic process can be disturbed by downregulated expression or decreased activities of carriers and enzymes for the TCA cycle and fatty acids β-oxidation. (**B**) OXPHOS. Electrons are extracted by complex Ⅰ and II from reducing equivalents, and move through ETC complexes, pumping protons into the IMS to generate MMP. MMP in turn drives proton flow back into the mitochondrial matrix through complex V, releasing this energy to generate ATP. Mitochondrial toxicants can reduce the expression and/or activity of ETC complexes, uncouple ETC from ATP synthesis, and impair MMP. (**C**) Redox homeostasis. mtROS produced in physiological state can be cleared by series antioxidant enzymes, such as GSH, SOD, and CAT. Drugs with mitochondrial toxicity can overproduce mtROS by inhibiting ETC complexes (especially complex Ⅰ and III) or decreasing the levels or activity of antioxidant enzymes, or there may be a combination of both mechanisms. (**D**) Map of mtDNA. The mitochondrion possesses its own genome, mtDNA, within the matrix, which can be replicated, transcribed, and translated into some of the MRC complexes. mtDNA, topoisomerase II for mtDNA repair, and DNA polymerase for mtDNA replication are all reported targets for toxicants. (**E**) mPTP. mPTP is a channel whose components have not been fully elucidated. The normal closed state can be triggered into an open state by a series of stresses, especially Ca^2+^ overload and oxidative stress. mPTP opening induces cytochrome c releasing into the cytoplasm, resulting in the initiation of apoptosis. (**F**) MQC. Defective mitochondria can be regulated by MQC, mainly including mitochondrial biogenesis and mitophagy. Damaged mitochondria can be cleared by mitophagy though ubiquitin-dependent or -independent pathways. In cardiomyocytes, ubiquitin-dependent pathway (Pink1-Parkin-mediated mitophagy) is induced by MMP depolarization, while the ubiquitin-independent pathway can be directly induced by LIR containing mitophagy receptors located on OMM in cardiomyocytes. Energy depletion after mitophagy activates genome-encoded transcriptional elements, which directly express mitochondrial proteins or regulate mtDNA to express related proteins for the assembly of new mitochondria. Toxicants may influence mitophagy or biogenesis to disturb MQC. Abbreviations: ANT: adenine nucleotide transporter; APAF: apoptotic peptidase activating factor; BNIP3: BCL2 interacting protein 3; C: cytochrome c; CAT: catalase; CPT: carnitine palmitoyltransferase; Complex I: NADH dehydrogenase; Complex II: succinate dehydrogenase; Complex III: cytochrome c reductase; Complex IV: cytochrome c oxidase; Complex V: ATP synthase; CypD: cyclophilin D; ERR: estrogen-related receptor; ETC: electron transport chain; FAs: fatty acids; FADH_2_: flavin adenine dinucleotide. FUNDC1: FUN14 domain-containing protein 1; FKBP8: FK506 binding protein 8; GPxs: glutathione peroxidase; GSH: glutathione; GSSG: glutathione disulfide; IBM: inner boundary membrane; IFMs: interfibrillar mitochondria; IMM: the inner mitochondrial membrane; IMS: intermembrane space; LC3: light chain 3; LDH: lactic dehydrogenase; LIR: LC3-interacting region; MFN1/2: mitofusin 1/2; MMP: mitochondrial 6membrane potential; MPC: mitochondrial pyruvate carrier; mPTP: mitochondrial permeability transition pore; NADH: nicotinamide adenine dinucleotide; NRFs: nuclear respiratory factors; OMM: outer mitochondrial membrane; OXPHOS: oxidative phosphorylation; PCMs: primary cardiomyocyte; PDH: pyruvate dehydrogenase; PGC-1α: peroxisome proliferator-activated receptorγ (PPARγ) coactivator 1α; Q: coenzyme Q; SOD: superoxide dismutase; SOD1: Cu/ZnSOD, copper- and zinc-dependent SOD; SOD2: MnSOD, manganese-dependent SOD; SSMs: subsarcolemmal mitochondria; TCA cycle: tricarboxylic acid cycle; TPx: thioredoxin peroxidase; UCP: mitochondrial uncoupling proteins; VDAC: voltage-dependent anion channel.

The heart consumes about 6 kg of ATP per day, which is mainly generated through mitochondrial OXPHOS from the catabolism of lipids and carbohydrates [133,134,135]. Glucose, lactate, and fatty acids are oxidized in the mitochondrion and produce a common end product (i.e., acetyl-CoA), which then goes through eight enzymatic steps of the Krebs cycle, where electrons are extracted from TCA intermediates in the form of reducing equivalents (nicotinamide adenine dinucleotide (NADH) and flavin adenine dinucleotide (FADH_2_)) (Figure 2A). The OXPHOS system links substrate oxidation to ATP generation (Figure 2B). It is made up of mitochondrial respiratory chain (MRC) complexes, including NADH:ubichinone oxidoreductase (NADH-dehydrogenase, complex I), succinate dehydrogenase (SDH, complex II), cytochrome c-reductase (complex III), cytochrome c oxidase (complex IV), and ATP synthase (complex V) [136]. Using NADH as a substrate for complex I and succinic acid for complex II, the MRC complexes transfer electrons from NADH and FADH_2_ to convert O_2_ to water (complex IV). The energy liberated during this mitochondrial electron transport is used to establish a highly electronegative potential (−140 to −180 mV), termed the MMP, across the IMM by extruding protons at complexes I, III, and IV into the IMS. When intracellular ADP is high, protons are channeled back into the matrix through the F0 portion of ATP synthase, fueling the conversion of ADP into ATP by the F1 portion of this enzyme. This process is tightly regulated, with close coupling of electron transport, membrane potential, and ATP synthesis. ATP is then moved from the mitochondrial matrix to the cytoplasm by the adenine nucleotide transporter (ANT), finally allowing energy to be available for cellular activity [137]. Substrate catabolism and OXPHOS are central to the energy homeostasis of mitochondria, and hence critical for cardiomyocyte functions. 

Many drugs are known inhibitors of the MRC, interfering with one or more of the complexes (Table 3). Inhibition can be caused by directly inhibiting the activity of MRC complexes. For example, zoniporide [64], naproxen [60,138], dronedarone [139], and mubritinib [140] inhibit complex I; propranolol and atenolol disrupt complex II [119]; celecoxib suppresses complex IV [14]; and As_2_O_3_ inhibits complex I, III, and IV [141]. OXPHOS may also be blocked by inhibition of the expression of MRC complexes, such as by mitoxantrone [100]. Additionally, uncoupling electron transport from ATP synthesis by tenidap [64] and nonsteroidal anti-inflammatory drugs (NSAIDs) (e.g., nimesulide, meloxicam, and acetylsalicylate) [142] is yet another way to inhibit OXPHOS. Lipophilic drugs can damage phospholipids on the IMM, especially cardiolipin [143], or activate the mitochondrial permeability transition pore (mPTP), which are mechanisms [144,145] that lead to pathologic uncoupling of respiration [72]. Bupivacaine has been suggested to interact with phospholipids in the IMM, which often result in increased membrane permeability, electron transport chain disruption, and calcium accumulation [146]. These inhibitory mechanisms of MRC complexes may cause a series of deleterious consequences. Firstly, OXPHOS inhibition can results in MMP dissipation and diminishment, or even abolishment, of ATP production [147]. Another important consequence of MRC inhibition is the secondary impairment of mitochondrial β-oxidation and the TCA cycle. Furthermore, blocking the flow of electrons favors reactive oxygen species (ROS) overproduction, leading to oxidative stress [148,149,150]. The majority of drugs with known mitochondrial liabilities display impairment of MRC function, highlighting it as a key indicator of mitochondrial health. The drugs listed in Table 4 are reported to downregulate the expression of proteins or to directly inhibit enzyme activity in FA oxidation and the TCA cycle.

### 2.3. Mitochondrial ROS (mtROS)

Mitochondria are the center of cellular aerobic metabolism and are thus also the major source of cellular ROS [191]. Electrons leaked from various sites (mainly complex I and III) of the MRC react with O_2_ and subsequently form a series of mtROS (Figure 2C) [191,192]. Nine of the eleven types of ROS are found in mitochondria [193]. In addition, studies revealed that extra-mitochondrial ROS could transduce signals into mitochondria and induce the production of mtROS. mtROS can be partly eliminated through antioxidant enzymes and antioxidant molecules, as well as glutathione and thioredoxin [194]. Oxidative stress is induced in response to a decreased level of antioxidant enzymes and increased production of ROS. Mitochondria, while being a primary source of ROS themselves, also suffer from ROS-mediated damages caused by peroxidation of macromolecules including proteins, lipids, and DNA [135,195,196,197]. Therefore, the balance between mtROS production and elimination is critical for mitochondria.

The balance of mtROS can be destroyed by toxic drugs decreasing antioxidant enzyme levels, inhibiting antioxidant enzyme activity, or producing mtROS (Table 5). The mitochondrial toxicity of some drugs, including cisplatin [112], azidothymidine [198], cannabinoids, glycosides [36], and pioglitazone [122], seems to be mediated by increases in the production of free radicals. DOX not only induces ROS production by Fe^2+^/Fe^3+^ cycling, but also inhibits antioxidant enzymes, including glutathione, glutathione peroxidase, and glutathione reductase [199,200]. Furthermore, ROS levels are elevated when OXPHOS complexes are inhibited [201,202] by drugs such as As_2_O_3_ [141]. Such free radicals can directly cause a diverse range of macromolecular damage, resulting in mitochondrial dysfunction. For example, ROS produced by DOX peroxidates cardiolipin, inducing cytochrome c release [188]. Hence, excessive mtROS production is a very common mechanism of mitochondrial dysfunction induced by mitotoxicants.

### 2.4. Replication, Translation, and Transcription of mtDNA

One cardiomyocyte contains up to 6000 mitochondria, each equipped with its own copies of mtDNA. Maintaining the stability, proper replication, transcription, and translation of mtDNA are critical for mitochondrial health [195]. The mtDNA encodes for 2 rRNAs, 22 tRNAs, and 13 proteins, which serve as essential subunits of the MRC complexes (Figure 2D) [78]. mtDNA is characterized by high gene density without introns or only separated by single noncoding nucleotides, thus requiring great accuracy to ensure the functionality of the resulting transcripts. mtDNA is transcribed at a high rate, especially in the highly energetically active heart [212]. The majority of the subunits and proteins in mitochondria are encoded by nDNA, whereas mtDNA only encodes 13 polypeptides of MRC complexes I, III, IV, and V. Translation of these 13 genes is accomplished via the mitochondria’s transcription and translation machinery, which comprises both nuclear-encoded protein factors, mitochondrial encoded RNA components, and mitoribosomes [213]. Critically, unlike nuclear DNA, mtDNA is not tightly packed into nucleosomes and proximal to ROS production sites [214], rendering it particularly susceptible to toxicants.

mtDNA can be interfered with by the inhibition of mitochondrial DNA polymerase and topoisomerase II (Table 6). DOX [158], mitoxantrone [215], and daunorubicin [130,207] inhibit DNA topoisomerase II β and disturb mtDNA stability and expression. DOX also decreases mtDNA and mtDNA-encoded subunit COX I in complex IV [96]. In addition, antiviral agents, such as zalcitabine, didanosine, and stavudine are specific inhibitors of the mitochondrial DNA polymerase-gamma, and therefore impair mtDNA replication [216]. It was later observed that nucleoside reverse transcriptase inhibitor-mediated mitochondrial toxicity can also occur as a result of direct inhibition of mtDNA-encoded protein synthesis [217,218]. Furthermore, antibiotics impair mtDNA-encoded protein synthesis due to the structural similarity between bacterial and mitochondrial ribosomes [219]. The mechanisms that deplete mtDNA-encoded protein levels eventually lead to decreases in ATP levels [202,220]. Clearly, mtDNA stability and expression are common targets of mitotoxic drugs, including anthracyclines, antiviral agents, and antibiotics. 

### 2.5. Mitochondrial Membrane Potential (MMP) and mPTP

MMP is both a chief function and key sentinel of the mitochondrial network, indicating the functional metabolic status of mitochondria. Through the establishment of MMP, ADP and inorganic phosphate (Pi) converts to ATP. Besides ATP generation, MMP is broadly required for both ion homeostasis and protein import into the mitochondrial network [221]. MMP dissipation can be dependently or independently induced by mitochondrial permeability transition pore (MPTP) opening [222]. Mitochondria are master regulators of cell survival. Ca^2+^ overload, excessive ROS production, MMP dissipation, fatty acid, and adenine nucleotide pool depletion have all been reported to trigger mPTP opening [223,224]. The opening of mPTP, a non-selective pore, is defined as a sudden increase in the permeability of IMM for small molecules and ions (<1.5 kDa), leading to cellular apoptosis and the occurrence and development of cardiovascular diseases [224,225]. However, the precise molecular composition of the mPTP is currently unknown [223]. ATP synthase, ANT, and cyclophilin D (CypD) have all been recognized as key components of the mPTP (Figure 2E) [226,227,228]. Normal MMP and closed mPTP are essential to healthy mitochondrial respiration and are therefore sentinels of mitochondrial impairment and cell survival.

Drugs inducing cardiotoxicity by targeting mitochondria invariably proceed to MMP collapse and mPTP opening (Table 3). Antineoplastic agents, including DOX [73,81,164], As_2_O_3_ [84,229], and imatinib [91]; β adrenergic receptor blockers, such as propranolol and atenolol [119]; antiarrhythmic drugs dronedarone and amiodarone [139]; antibiotics erythromycin and clarithromycin; NSAIDs such as naproxen, diclofenac, and celecoxib [60]; and diabetes drug pioglitazone [122] have all been reported to cause these harmful effects. mPTP opening and MMP decrease consequently induce loss of respiratory control and imbalance in ATP production, and loss of mitochondrial components such as ATP, NAD+, and glutathione, leading to water accumulation in the matrix, which causes mitochondrial osmotic swelling, IMM unfolding, and the rupture of the OMM [230,231]. As a gatekeeper of cellular death, the opening of the mPTP eventually results in the release of pro-apoptotic proteins from the IMS into the cytosol, thus stimulating both caspase-dependent and caspase-independent apoptosis [202]. Due to the universality of MMP collapse and mPTP opening induced by mitotoxicants, evaluating their status has become one of the most basic and routine tests in the assessment of drug-induced mitochondrial dysfunction.

**Table 6:** Drugs affecting mitochondrial carriers and mtDNA, their clinical manifestations, and relevant in vitro and in vivo studies.

Modules	Alterations	Pharmacology	Drugs	Clinical Manifestations	C_max_	Models	Dose	Time	References
Carrier	Downregulation of CPT I expression	Alkylating agent	Cyclophosphamide	HMC, CMP	143 μM	Male Wistar rats (IP)	200 mg/kg	10 d	[189]
Carrier	Downregulation of CPT I expression	Anesthesia	Propofol	HF, arrhythmia	30.13 μM	HiPSC-CMs	10 µg/mL	48 h	[163]
Carrier	Downregulation of CPT I expression	TKIs	Sunitinib	Decreased LVEF, QT prolongation, TdP, hypertension, HF, CMP	0.25 μM	Rats (oral)	25 mg/kg/d	28 d	[173]
Carrier	Inhibition of CPT1 activity	Anti-arrhythmic drug	Dronedarone	AF, HF	0.15–0.26 μM	Isolated rat heart mitochondria	IC_50_ = 40 µM	20 min	[139]
Carrier	loss of carnitine	Co-catalyst	Pivalic acid	CMP					[232]
Carrier	Inhibition of ANT	NSAIDs	Diclofenac	Hypertension, arrhythmias	7.9 µM	Submitochondrial particles	314 nM/mg protein diminished 76%		[142]
			Nimesulide			Submitochondrial particles	259 nM/mg protein diminished 60%		[142]
Carrier	Inhibition of ANT	NRTIs	Zidovudine	CMP	4 μM				[233]
mtDNA	Inhibition of mitochondrial DNA polymerase	NRTIs	Zidovudine	CMP	4 μM	Cardiac DNA pol-γ	1 µM		[234]
mtDNA	Inhibition of topoisomerase II	Anthracyclines	DOX	CHF, decreased LVEF, ST, myocarditis, CMP	15.3 μM	-	-	-	[235]
			Daunorubicin	CMP, MI, CHF, VA, pericarditis, myocarditis	89 μM	-	-	-	[207]
			Idarubicin	CMP, MI, CHF, VA, decreased LVEF	23.22 μM	-	-	-	[207]
mtDNA	Inhibition of topoisomerase II	Chemotherapeutic agents	Mitoxantrone	CHF, CMP, decreased LVEF, arrhythmia	3.3 μM	-	-	-	[215]
mtDNA	mtDNA content decreasing	Anthracyclines	DOX	CHF, decreased LVEF, ST, myocarditis, CMP	15.3 μM	Male Wistar rats (IV)	1 mg/kg/w	7 w (started at 11 w, observed at 48 w)	[96]
mtDNA	mtDNA content decreasing	TKIs	Regorafenib	MI; hypertension	8.08 μM	H9c2	5 μM	72 h	[90]

Abbreviations: NRTIs: nucleoside reverse transcriptase inhibitors; NSAIDs: nonsteroidal anti-inflammatory drugs; TKIs: tyrosine kinase inhibitors; ANT: mitochondrial ADP/ATP transport; TCA: tricarboxylic acid; DOX: doxorubicin; CHF: congestive heart failure; LVEF: left ventricular ejection fraction; HF: heart failure; TdP: torsades de pointes; MI: myocardial infarction; AF: atrial fibrillation; CMP: cardiomyopathy; ST: sinus tachycardia; HMC: hemorrhagic myocarditis; VA: ventricular arrhythmia; HA: heart attack; IP: intraperitoneal; IV: intravenously; BID: twice daily; w: week; d: day; h: hours.

### 2.6. Mitochondrial Carriers

Mitochondrial function (including the TCA cycle; fatty acid oxidation; OXPHOS; amino acid degradation; biosynthesis of amino acid, iron sulfide, urea, heme, and cholesterol; and dissipation of proton gradient for heat production) relies on the exchange of a very diverse set of solutes and metabolites across the IMM and OMM. Mitochondrial carriers located on the IMM ensure that massive transport processes can occur between the mitochondrial matrix and the cytoplasm. These specific carriers are a superfamily of nuclear-encoded proteins including the solute carrier (SLC) family, the sideroflexin family, the mitochondrial pyruvate carrier (MPC1/2), ATP-binding cassette transporter (ABCB) isoforms, and splice variants of other solute carriers, as well as recently identified mitochondrial amino acid carriers [236]. Consequently, mitochondrial carriers are fundamental for various mitochondrial functions.

Mitochondrial toxicity can occur as a result of inhibition of carriers, such as adenine nucleotide translocator (ANT) for exchanging ADP in for ATP out [237,238,239] and carnitine palmitoyltransferase (CPT) I and II for fatty acyl-CoA transfer into the matrix (Table 6) [202]. Zidovudine impairs ANT activity, which is one of the biochemical processes responsible for cardiomyopathy [233]. Inhibiting the export of ATP causes MRC inhibition, compromising cell function due to energy deficiency [216]. Pivalic-acid-induced cardiomyopathy is the result of CPT activity inhibition, which is responsible for fatty acid oxidation in mitochondria [36,232]. In addition, sunitinib significantly decreased the level of CPT I expression [173]. In addition to ANT and CPT, other carriers potentially targeted by drugs are worth investigation. 

### 2.7. Mitochondrial Quality Control (MQC)

Cardiomyocytes require healthy mitochondrial homeostasis to provide sufficient ATP for maintaining the pump function of the heart. Mitochondrial structure and function are tightly regulated by the MQC system, which is fundamental for sustaining mitochondrial bioenergetics demand and metabolic functions [240]. MQC is a series of processes comprising quality control of mitochondrial proteins, mitochondrial biogenesis, mitochondrial dynamics (fission and fusion), and mitophagy (Figure 2F) [241]. MQC repair or remove dysfunctional and damaged mitochondria; promote mitochondrial regeneration; improve mitochondrial biochemical processes and signaling transduction; maintain mitochondrial morphology, quantity, and function; and promote cell survival [242]. The vast majority of unrepaired damaged proteins are removed through the cytosolic ubiquitin/26S proteasome system (UPS), which has been shown to be crucial in the quality control of mitochondrial proteins [243]. In cardiomyocytes, mitophagy pathways include ubiquitin-dependent pathways, such as the PTEN-induced kinase 1/E3 ubiquitin ligase parkin (PINK1/Parkin) pathway, and ubiquitin-independent pathways, such as the BCL2 and adenovirus E1B 19 kDa-interacting protein 3 and BNIP3-like (Bnip3/Nix) pathway [244]. Due to its critical role in maintaining the mitochondrial network, MQC has recently been found to be vulnerable to unfavorable factors, including disease and toxicants [245].

UPS, mitochondrial biogenesis and dynamics, and mitophagy are reported targets for drug-induced cardiotoxicity (Table 2). Trastuzumab inhibits mitochondrial biogenesis, possibly through Her2-dependent estrogen-related receptor alpha activation [246]. Etoposide [169], zidovudine [131], and remdesivir were reported to disrupt mitochondrial dynamics, promoting mitochondria fragmentation [86]. Mitophagy mediates the clearance of damaged mitochondria with excessive mtROS marked by MitoSOX or/and decreased MMP indicated by JC-1 [247,248]. Mitophagy can be visualized in vivo using mitophagy-associated fluorescence proteins, such as mt-keima, mito-QC, and RFP/GFP-LC3 [249]. Colocalization of mitochondria (marked by MitoTracker or mitochondrial-specific fluorescent antibody) and autophagosomes (indicated by GFP-LC3) or lysosomes (dansylcadaverine, LysoTracker, or lysosome-specific fluorescent antibody) under fluorescence microscope, as well as immunoblotting of Parkin, LC3II/I, ubiquitin, Atg5, Beclin1, and p62 are widely used methods for mitophagy detection in vitro [247,250,251,252,253]. Based on these methods, sunitinib and sorafenib were shown to impair mitophagy via inhibition of ribosomal S6 kinase and AMPK. As_2_O_3_ induced parkin-dependent UPS activation [129]. Excessive mitophagy induced by the Parkin/PINK pathway contributed to DOX-induced toxicity [127,128]. On the other hand, mitigation of BNIP3-dependent mitophagy by aconitine induced cardiomyocyte damage [121]. Recently, increasing numbers of drugs interrupting MQC have been reported, indicating MQC impairment as a potentially critical criterion for mitotoxicant identification.

### 2.8. Other Mitotoxicants and Their Targets

Most mitochondria-related toxins and their targets are searchable in MitoTox [51]. In addition to the above-mentioned targets for mitotoxicity, several other toxicological mechanisms have been implicated in mitochondrial dysfunction but require further interrogation in cardiomyocytes. Ion channels and transporters on the IMM are associated with redox regulation and electrical and contractile dysfunction of cardiomyocytes [254]. H^+^/Na^+^ antiporters can be inhibited by amiloride analogs [255]. Inner membrane anion channels that are permeable to a variety of anions and anionic metabolites can be blocked by amiodarone, dibucaine, propranolol, amitryptiline, and clonazepam [254,256]. However, not all of these ion channels and transporters have been confirmed as a mechanism mediating mitochondrial toxicity in cardiomyocytes. The mitochondrial unfolded protein response (mtUPR) that is induced upon stress serves an important protective role in the heart by ameliorating mitochondrial dysfunction [257]. Although activated mtUPR resists statin toxicity in C. elegans [258], there is still a lack of evidence for the association between mtUPR and mitochondrial toxicants, especially in cardiomyocytes. Many other emerging molecular processes are gaining attention as mechanisms underlying mitochondrial dysfunction, and thus could be potential mediators of toxicity. Protein post-translational modifications (PTMs) were found to contribute to heart failure progression by regulating mitochondrial function [259]. Of the many proteins regulated by PTMs, mPTP is gaining attention as a potential mechanism underlying mitochondrial dysfunction and has as many as 55 PTM sites [260]. It is, therefore, possible that PTM of mPTP may contribute to mitochondrial toxicity in the heart. 

## 3. Limitations of Current Preclinical Models for Assessing Cardiotoxicity

The successful development of a new drug takes about 14 years, at an average cost of USD 403 million, with roughly one-third spent on preclinical studies and the rest on clinical trials [261,262]. As a leading cause of attrition, drug-induced toxicity appears at all stages of drug development [263]. Even when drugs pass through the series of preclinical evaluations for drug safety, only 11% make it through clinical studies [264]. More strikingly, 462 marketed drugs were withdrawn due to ADRs from 1953 to 2013 [264]. Among all types of drug toxicity, cardiovascular toxicity constitutes 30% of all organ-toxicity-caused attritions [265], as well as 14% of all drug-toxicity-related withdrawals. Still, there remains 15–35% of drugs in the market with cardiovascular ADRs, which may contribute to the high and still increasing morbidity and mortality rates of CVDs, which claimed over 17.6 million lives in 2016 alone worldwide [266]. Therefore, drug-induced cardiac toxicity has caused serious financial losses for the pharmaceutical industry, as well as harm to patients’ well-being. The high rate of cardiotoxicity-related drug attrition, withdrawal, and ADRs are attributed to the insufficient cardiac safety evaluation system. Given that a 10% improvement in toxicity prediction at the preclinical stage could save $100 million per drug [263], improvements in early cardiotoxicity identification may have a profound impact on costly late attrition. It also helps to avoid unexpected ADRs that may threaten patients’ lives [263]. 

### 3.1. Limitations in the Current Workflow of Cardiac Safety Testing

Currently, drug-induced arrhythmia, especially QT prolongation leading to life-threatening complications including torsade de pointes, ventricular tachycardia, and sudden cardiac death, is the predominant concern during drug development. Arrhythmia can be induced by a wide range of drug classes including both non-antiarrhythmic drugs and, paradoxically, antiarrhythmic drugs [267]. In order to identify proarrhythmic drugs, the nonclinical ICH S7B (in vitro human ether-a-go-go-related (hERG) current and in vivo QT assays) and clinical ICH E14 (thorough QT study) guidelines were issued in 2005, which effectively precluded drugs with QT prolongation effects from further development [268]. According to these guidelines, in vitro hERG current measurement is mostly performed in immortalized heterologous cell lines stably expressing hERG channels using the patch-clamp technique, while in vivo QT assays are performed in animal models via electrocardiography. However, QT prolongation is not a robust surrogate for arrhythmogenic risk, and drugs blocking the hERG current do not always cause arrhythmias, resulting in nearly 60% of promising compounds being mistakenly eliminated during development [269]. In August 2020, ICH released an updated guideline combining S7B and E14 Questions and Answers (Q&As) to define an appropriate and efficient assessment of drug-induced corrected QT interval prolongation. The comprehensive in vitro proarrhythmia assay (CiPA) is a new strategy to determine the arrhythmogenic effects of drugs by evaluating a variety of cardiac repolarization-related currents in heterologous expression systems, reconstructing cardiac electrophysiologic activity in silico, followed by validations in human induced pluripotent-stem-cell-derived cardiomyocytes (hiPSC-CMs) and supplementation with data from phase I clinical trials. Although these guidelines have proven effective at pro-arrhythmic risk assessment, the complexity of drug-induced cardiotoxicity goes far beyond abnormal heart rhythm. Mitochondrial toxicity is increasingly implicated in drug-induced cardiotoxicity. Over 50% of clinical drugs causing cardiovascular adverse events, eliciting black box warnings from the U.S. Food and Drug Administration (FDA), are caused by mitochondrial liabilities [147]. However, mitochondrial toxicity testing has not been incorporated into routine safety testing procedures during drug development.

Another caveat of the current cardiac safety evaluation system is the failure to identify hidden cardiotoxicity. Hidden cardiotoxicity is a type of toxicity that goes undetected in healthy individuals, and only reveals itself under disease conditions [262]. Diseased hearts are loaded with function alterations involving ion channels, mitochondria, and electro-mechanical coupling, and are often more vulnerable to drugs. For example, cardiac arrhythmias may only be revealed in preclinical models of cardiovascular disease (e.g., myocardial infarction) [270]. This could be one of the reasons for the high rate of cardiotoxicity-related drug attrition, withdrawal, and ADRs. Although these aspects are important, given the focus of this article, we will only discuss viable measures to detect mitochondrial toxicity preclinically.

### 3.2. In Vitro Models for Cardiac Toxicity Assessment

Understanding the advantages and limitations of cardiac models is critical for proper cardiac risk assessment. To assess and confirm mitochondrial toxicity, physiological relevance ranked from high to low is as follows: human data, animal models, organ models, cell models, and organelle models. However, animal and organ models are not only low-throughput, expensive, and time-consuming, but also frequently incapable of directly reporting mitochondrial impairment. Isolated mitochondria provide a useful in vitro experimental system for mechanistic analysis, with the advantage of allowing extensive control over experimental conditions, such as measuring toxic effects on mitochondrial oxygen consumption without interference from other physiological processes [271]. Mitochondria subtypes, including SSMs and IFMs, can be either jointly or individually [272,273] isolated from cultured cardiomyocytes and heart tissues. However, their low yield, lack of physiological environment, and biased extraction of healthier ones from the sample [271] make isolated mitochondria not suitable for HTS. In contrast, intact cardiomyocytes exhibit several advantages over other models for evaluating drug-induced cardiotoxicity. In contrast with whole-heart preparations and tissue slices, cardiomyocyte cultures rule out signal contamination from other cell types, thereby identifying cell-type-specific toxicity. Unlike isolated mitochondria, cardiomyocytes provide a more physiologically relevant cellular environment, including materials for mitochondrial import and export, normal subcellular arrangements and structures of mitochondria, all mitochondrial subtypes with specific subcellular localizations, and so on. At present, three cell models are used according to the new E14/S7B draft guideline, including cell lines stably expressing hERG channels, hiPSC-CMs, and human primary cardiomyocytes (hPCMs). Heterologous hERG-expressing cell lines, while useful for single-channel screening, do not recapitulate the complex electrical activities seen in cardiomyocytes. By contrast, intact cardiomyocytes are more physiologically relevant for evaluating drug-induced cardiotoxicity. Currently used cardiomyocyte models include H9c2 cardiomyoblasts, stem-cell-derived cardiomyocytes, and primary cardiomyocytes (PCMs). 

#### 3.2.1. H9c2 Cardiomyoblasts

The H9c2, derived from the ventricular part of the embryonic BDIX rat heart [274], is an immortalized myoblast cell line used as cardiomyocytes due to its biochemical, electrophysiological, and hormonal signaling properties [274,275]. By sequential selective passaging and culturing with all-trans retinoic acid and 1% serum media, they may overexpress L-type calcium channels, mimicking the adult cardiac muscle phenotype [276,277]. H9c2 cardiomyoblasts have been used for mitochondrial toxicity assessment of DOX, and demonstrated increased mitochondrial swelling [278], mtROS [279], mitochondrial fission [280], decreased MMP [281], and OCR and ATP production [282], whereas trastuzumab led to increased mtROS and decreased MMP [185]. Treatment of H9c2 cells with tyrosine kinase inhibitors, such as imatinib, sorafenib, and sunitinib, resulted in mitochondrial swelling, cristae loss, MMP reduction, inhibition of MRC complexes, superoxide accumulation, and cellular GSH depletion [283,284,285]. Similar mitochondrial impairments were also observed with other toxicants, such as As_2_O_3_ [286] and simvastatin [151]. H9c2 was utilized in HTS to identify compounds potentially conferring protection from DOX-induced damage [287]. However, H9c2 cardiomyoblasts have a lower predictive value than hESC-CMs with regard to ATP levels, MMP, Ca^2+^ mobilization, and endoplasmic reticulum integrity with therapeutic concentrations of toxic drugs [288]. Furthermore, drug responses may vary with differentiation state [289,290]. Ultimately, H9c2 cells are not of human origin, with differences including mitochondrial content and metabolism potentially affecting their translational capability, thus limiting their use as a model targeting mitochondrial toxicity [69]. 

#### 3.2.2. Stem-Cell-Derived Cardiomyocytes

Stem-cell-derived cardiomyocytes, including human embryonic stem-cell-derived cardiomyocytes (hESC-CMs) and hiPSC-CMs, are derived from blastocysts or reprogrammed somatic cells, respectively, with a series of differentiation processes [264]. They resemble in vivo cardiomyocytes in terms of ultrastructure [291], electrophysiology, and contraction [3], and are widely used as surrogates for native human cardiomyocytes [3], providing a promising platform for cardiotoxicity assessment [292]. However, hESC-CMs are limited by ethical concerns and regulatory restrictions. hiPSC-CMs, while circumventing these problems, face other challenges, the most prominent of which is immaturity [293]. Fortunately, many techniques are being developed that aim at enhancing cardiomyocyte maturation [294,295,296,297,298], thus improving drug response [299,300,301]. Other efforts are directed at the mass production of hiPSC-CMs for screening purposes. Approximately 1.5–2.8 billion cardiomyocytes per bioprocess can be generated by two-dimensional (2D) and 3D culture systems [302,303], and this number can be increased a hundred-fold through the inhibition of the glycogen synthase kinase-3β (GSK-3β) pathway [301], meeting the demands of HTS [304,305]. Downstream screening, and advances in computational methods, such as artificial intelligence (AI) algorithms, are being developed to more accurately define endpoints, as has already been implemented for Ca^2+^ transients [306] and cardiomyocyte structure [307]. hiPSC-CMs, a human-based cardiomyocyte model with patient- and disease-specific characteristics, are versatile tools for phenotypic or target-based screening in lead compound discovery, as well as preclinical arrhythmia detection as required by CiPA. 2D hiPSC-CMs achieved 90% sensitivity, 74% specificity, and 82% accuracy in detecting drugs blocking ion channels and contraction [308]. Similarly good performance was also reached when profiling drugs using metabolic and viability endpoints [309]. Toxicities induced by anticancer therapies, including anthracyclines and tyrosine kinase inhibitors, were also successfully recapitulated in 2D hiPSC-CMs [301]. Chronic cardiotoxicity usually emerges between one month and decades after administration of treatment doses and might only be discovered during post-market monitoring [8]. Cardiotoxicity studies performed with single and relatively short exposure periods (up to 48 h) do not reflect the true mechanisms of chronic cardiotoxicity [310]. Given that delayed-onset cardiotoxicity may take longer to detect [21], long-term cultured cells with long-term recording are required [311]. hiPSC-CMs can beat spontaneously with stable baseline functions for months [312,313], rendering them more suited for assessing chronic toxicity. Notably, hiPSC-CMs have been widely used as a cellular model for evaluating the chronic effects of anticancer drugs and nucleoside analogs [23,293,295,297,298]. However, there is increasing awareness of the differences between hiPSC-CMs and their primary counterparts (i.e., hPCMs) concerning metabolism, structure, and function [293,314]. hiPSC-CMs remain similar to other cell lines in terms of their mitochondrial morphology, distribution, and function. In hiPSC-CMs, mitochondria occupy only about 5% of the cell volume and are located around the nucleus. Morphologically, they assume a rounded shape with poor cristae. Metabolically, they mostly rely on glucose for ATP production (~85%) [314]. All of these result in resistance to mitochondrial toxicity measurements [315].

#### 3.2.3. hPCMs

PCMs are directly harvested from the native tissue and are considered to possess all properties of normal cardiomyocytes in the heart. Therefore, they are well suited for pharmacological evaluation of cellular morphology, function (e.g., electrophysiology, calcium handling, contraction), and subcellular structures, such as mitochondria [316,317]. Additionally, they are exceptional tools with regard to disease modeling, because they can be directly isolated from target animals or patients, eliminating the need for external manipulations, as is routine with all other biological models [3]. PCMs can be isolated from the embryonic, neonatal, or adult stages of animals and humans. However, owing to species differences, PCMs derived from humans and animals vary significantly in their functional and molecular characteristics [318]. hPCMs probably bear the highest degree of resemblance to native cardiomyocytes and are thus well suited for cardiotoxicity assessment. Preserving the donor’s genetic background, clinical manifestation, and medical history is a particular advantage of hPCMs, as drugs may induce unexpected effects in an old, diseased, and susceptible heart that is hidden in the healthy one [262]. Most of the current cardiotoxicity detection platforms for measuring contractility, calcium transient, membrane potential, and mitochondrial functions in hiPSC-CMs can also be applied to hPCMs [293,319]. Twenty-six inotropes (17 positive, 9 negative) were identified in adult hPCMs based on contractility transients [320]. It is worth noting that compared with hPCMs, hiPSC-CMs exhibited higher rates of false-positive and negative results for 33 multi-ion channel-blocking drugs [321]. Except for limited availability and technical challenges in handling, hPCMs are ideal tools for high-throughput assays examining the effects of compounds on mitochondria, due to their abundance, subtype distribution, shape, and substrate utilization [299,305,308]. Mitochondria occupy 30% of the hPCM cell volume and are distributed between myofibrils, under sarcolemma, and at the two longitudinal poles of the nucleus. They are also more reliant on fatty acids (80%) as the metabolic substrate, whose oxidation process for ATP production is reliant upon mature mitochondria [314]. Based on higher mitochondrial content, an elevated ROS level after doxorubicin treatment in more mature cardiomyocytes was detected compared to immature cardiomyocytes [322]. However, despite recent progress in hPCM isolation [323,324,325] and culture [326,327], they have not been used for mitochondrial toxicity screening to date. 

#### 3.2.4. 3D Cardiomyocyte Models

3D cardiac models are a promising class of models in that they are of human origin, reflect in vivo cardiomyocyte physiology and function, comprise multiple cell types, are suited for evaluation of both acute and chronic toxicities, and are available in high-throughput format [328,329]. Cardiac spheroids are a type of self-assembled ball shape structure comprising one or multiple cell types. They have been used to verify the cardiotoxicity of DOX, sunitinib, verapamil, and quinidine at clinically relevant concentrations, assessed by cell viability, contractility, MMP, and endoplasmic reticulum integrity [283,317,318]. Currently, cardiac spheroids can be easily generated in microscale platforms, such as 96/384-well plates [300,330], to decrease the number of cardiac cells. Cardiac organoids are hollow 3D structures of relevant cardiac cells, including cardiomyocytes, endothelial cells, fibroblasts, and so on, in the presence of extracellular matrix proteins. Cardiac organoids resemble the human heart by exhibiting similar ultrastructure and physiology, including oxidative metabolism, force-frequency relationship, and calcium handling [298]. Transcriptomic analysis revealed that cardiac organoids share the highest degree of similarity with human adult myocardium compared with 2D, 3D hiPSC-CMs, and fetal myocardium [331]. Small-size engineered heart tissue platforms have also been described [332]. Measurements using cardiac organoids can also be conducted in a high-throughput manner and are compatible with the detection of electrophysiological abnormalities, contractile dysfunction, and structural toxicity [333,334]. More importantly, cardiac organoids showed drug evaluation results similar to the adult heart [316,323,324]. The reactivity of cardiac organoids induced by clinical compounds, including antibiotic, antidiabetic, and anticancer drugs, was shown to be more physiologically relevant compared with 2D-cultured hiPSC-CMs [335,336]. Based on a panel of eight metrics, cardiac organoids responded appropriately to pro-arrhythmic stimuli and effectively differentiated between high- and low-risk hERG-inhibiting compounds, meeting the critical demand in pro-arrhythmic cardiotoxicity prediction [337]. In addition to electrophysiology, cardiac organoids are also sensitive to drugs affecting cardiac contractility and can be applied in HTS format using a customized image acquisition workflow and optical flow analysis methods [329,335]. Structural parameters, including cell membrane permeability, MMP, endoplasmic reticulum integrity, and cellular viability, can be measured in cardiac organoids using high-throughput assays as well [332,334]. Therefore, 3D cardiomyocyte models hold great promise for cardiotoxicity screening. However, the technical challenges are still relatively high. As a 3D structure, organoids frequently present a necrotic core owing to the heterogeneous diffusion of nutrients. Similarly, drugs not evenly distributed by diffusion in the cardiac organoids also influence the accuracy of toxicity prediction [328]. Furthermore, the production of sufficiently large quantities and sufficient uniformity of generated organoids for high-throughput assays is a challenging task. Thus, additional work is needed to make these models available to the pharmaceutical industry. 

## 4. Proposed Preclinical Model of Cardiomyocytes for Assessment of Drug-Induced Mitochondria Toxicity

### 4.1. In Vitro Cell Culture for Cardiotoxicity Assays

H9c2 can be either self-differentiated or purchased from cell banks (e.g., American Tissue Culture Collection (Manassas, VA, USA) [185,279] and Cell Bank of the Type Culture Collection of the Chinese Academy of Sciences (Shanghai, China) [280,282]) and cultured with Dulbecco’s modified Eagle’s Medium (DMEM; Gibco) containing 10% fetal bovine serum (FBS; Gibco), 100 U/mL penicillin, and 100 μg/mL streptomycin. In addition, 2D hiPSC-CMs can be self-differentiated and cultured with RPMI + B27 with insulin [338,339], or purchased from biotechnology companies (e.g., Cellular Dynamics International (Madison, WI, USA) [308] and FUJIFILM Cellular Dynamics, Inc (FCDI, Madison, WI, USA) [309]), and maintained in culture according to their protocols. As for 3D cardiomyocyte models, hiPSC-CMs are the most common type of cardiomyocytes used in cardiac organoids. Other non-cardiomyocytes can be either induced from hiPSCs [332] or isolated from human tissue. Cardiac organoids are cultured with 50% cardiomyocytes maintenance medium and 50% endothelial basal medium when endothelial cells are included in organoids [300,329]. Alternatively, a 100% cardiomyocytes maintenance medium is used when cardiac organoids only consist of cardiomyocytes and fibroblasts [298]. hPCMs are isolated from human heart samples during surgical procedures such as coronary artery bypass surgery, valve replacement, and so on, and may be cultured with ACCITT3 culture medium [327].

The carbon source in the culture media of cardiomyocytes is one of the most critical determinants of reliable mitochondrial toxicity evaluation. Cells grown in media containing glucose may cause the ‘Crabtree effect’ [340], allowing high levels of glycolysis with minimal OXPHOS, and altered mitochondrial physiology. This artificial shift in metabolism undermines the effectiveness of HTS assays examining mitochondrial toxicity. On the contrary, when grown under conditions of low-glucose or glucose-free media with abundant oxygen supply, cardiomyocytes are forced to use OXPHOS for ATP production [340], exhibiting mitochondrial respiration comparable to in vivo conditions [341]. Cardiomyocytes, including H9c2 [283,284,285,342], hESC-CMs [140], and hiPSC-CMs [343], grown in galactose become susceptible to mitochondrial toxicants [344,345]. None of the H9c2 cells cultured in galactose survived troglitazone treatment, whereas those in high-glucose medium were unaffected 24 h post-treatment [346]. Owing to the 2–3 orders of magnitude higher sensitivity to various mitotoxicants with galactose culture [345], the differential sensitivities of glucose- versus galactose-grown cells were therefore used as an identifier of specific drug-induced mitochondrial impairment. Specifically, a ratio of half-maximal inhibitory concentration (IC_50_) of a drug, based on ATP production as a readout, in glucose- and galactose-grown cells (IC_50_ Glucose: IC_50_ Galactose), of >3 is taken as an indication of mitochondrial toxicity [345,346]. Mitochondrial liabilities for members of the biguanide family as well as certain antidepressants (nefazodone) were identified in this manner [347], and the approach has since found widespread use across the pharmaceutical industry. Of note, this method worked particularly well for inhibitors of ETC complexes I and III, but was useless for uncouplers [345] and other toxic effects, including mitochondrial ion channels inhibition and DNA damage [267]. 

### 4.2. Mitochondrial Target as Readouts in Cardiotoxicity Assays

#### 4.2.1. Mitochondrial Morphology, Structure

Mitochondria toxicity can be detected by its abundance, arrangement, and morphology alterations. Traditionally, these changes are detected by transmission electron microscopy [348], a method providing only a snapshot in a specific space and time. Nowadays, changes in mitochondrial dynamics can be visualized directly by high-content screening (HCS) in 96- and 384-well plates. Multiple fluorescent probes, mitochondria-tagged fluorescent proteins, or immuno-labelling with antibodies can indicate mitochondrial abundance, arrangement, morphology (e.g., swollen, punctate, etc.). Fluorescent probes, such as nonyl-acridine orange (NAO), measuring mitochondrial cardiolipin content, and Mito Tracker, which determines MMP, are used to characterize subpopulations of mitochondria by HCS [349]. Mito Tracker is retained in fixed mitochondria and is therefore compatible with antibody-based imaging [350]. Constructs expressing fluorescent proteins (i.e., GFP, RFP, YFP) fused with specific sequences are also used for mitochondrial analysis by targeting the OMM, IMM, or matrix [351,352]. Immuno-labeled antibodies targeting specific proteins, such as MRC complexes or TOM20 on the OMM, can also be used for HCS [353]. Systematic image analysis software now makes it possible to quantify mitochondria in cardiomyocytes in a high-throughput manner [307]. 

#### 4.2.2. Oxygen Consumption Rate (OCR)

Oxygen consumption, one of the classic end points of assessing the metabolic implications of drug treatment, provides direct information on the activity of OXPHOS. OCR is highly sensitive to perturbations in mitochondrial function [354]. Traditionally, OCR measurements on isolated mitochondria are performed using Clark-type oxygen electrodes [355,356]. Today, Seahorse Bioanalyzers represent a significant advance in OCR assessment, improving both throughput and sensitivity [357,358,359]. By orderly injection of chemical probes including oligomycin, FCCP, rotenone, and antimycin A, a series of readouts, including basal respiration, proton leak, non-mitochondrial oxygen consumption, maximal respiration, ATP production, spare respiratory capacity, and coupling efficiency, can be calculated to reveal OXPHOS damage. Reductions in OCR can result from altered control mechanisms (e.g., MMP decline), diminishments in the supply of reducing equivalents, inhibition of individual MRC complexes, or ANT inhibition. Therefore, the primary mechanism for such reduction needs to be identified as the next step. When necessary, the activity of individual complexes can be interrogated through the use of specific respiratory substrates and inhibitors [345,360].

#### 4.2.3. ATP

Determining cellular ATP levels is an effective and robust way to assess compound toxicity [361]. Since mitochondria are the sites for ATP production in cells, decreases in ATP levels indicate impaired mitochondrial function. During apoptosis, reductions in ATP are usually accompanied by decreases in the MMP. As a secondary measurement for mitochondrial function [362], ATP content is frequently used as an indicator of cellular viability in HTS [339]. ATP content can be measured by colorimetry, fluorescence, luminescence, or isotopes. Photoluminescence measurement on microplate readers is the most popular method at present [363,364,365,366], due to its superior detection sensitivity and operational convenience. A rhodamine-based spirolactam ATP sensor was developed to specifically monitor mitochondrial ATP in real time and has already been applied to human and mouse skin fibroblasts [367]. 

#### 4.2.4. Redox Homeostasis

The balance between the generation and neutralization of ROS is another important determinant of mitochondrial health. Therefore, the functional state of mitochondria can be reflected by detecting the level of ROS, especially mtROS [362]. ROS is adapted to HTS platforms with probes including MitoPY1 [365], MitoSox [368], and CellRox [369]. MitoSOX, a mitochondrially targeted fluorescent dye [370], is widely used for the measurement of O_2_ formation in active mitochondria. Another ROS probe, Amplex UltraRed, is oxidized by H_2_O_2_ to form a fluorescent product and is specifically used in monitoring H_2_O_2_ production [371,372]. Upon detection of aberrantly high ROS levels, dysfunction of the antioxidant system is frequently interrogated as a potential mechanism. For example, SOD activity can be determined by pyrogallol autoxidation, while glutathione levels can be assessed through its oxidation by 2-nitrobenzoic acid. Furthermore, downstream of ROS overproduction, ROS-mediated damage-induced peroxidation of macromolecules is also an indicator of mitochondrial toxicity. For example, the levels of malondialdehyde, one of the final products of polyunsaturated fatty acids peroxidation, can rise as a consequence of an increase in free radicals. Its reaction with thiobarbituric acid provides a colorimetric approach to evaluating lipid peroxidation [373].

#### 4.2.5. MMP

Assays using fluorescent probes to quantify disruption of MMP have been validated as an effective method for assessing mitochondrial toxicity and have been adopted for HTS [374]. Although cell models vary in their responses to mitochondrial toxicants, MMP is a steady criterion to indicate mitochondrial dysfunction [375]. MMP-dependent lipophilic and cationic dyes, including rhodamine 123, tetramethylrhodamine methyl (TMRM) [376], tetramethylrhodamine ethyl ester (TMRE), 5,5′,6,6′-tetrachloro-1,1′,3,3′-tetraethylbenzimidazolycarbocyanine iodide (JC-1), and JC-10 [375] are widely used to assess MMP [377]. The widely used probe JC-1 and its modified, water-soluble version, JC-9, accumulate in mitochondria MMP-dependently and exhibit a shift in emission wavelength from green (monomers) to red (J-aggregates), providing a readout of the potential difference across the IMM. Although MitoTracker MMP-dependently labels mitochondria [378], it is more generally used as a mitochondrion-specific probe to track mitochondria, for example in colocalization experiments with ROS indicators [379,380,381] or lysosomes indicators [382]. However, MMP quantification alone cannot distinguish whether such loss is due to inhibition of MRC complexes, uncoupling, or mitochondrial permeability transition, so complementary assays are required to determine the underlying causes [362].

### 4.3. High-Throughput Assessment of Mitochondrial Toxicity

Owing to the diverse range of drugs that can cause cardiac mitochondrial toxicity (Table 2, Table 3, Table 4, Table 5 and Table 6), and due to the varying degrees and types of toxic manifestations, it would be desirable to screen for such toxicity in a high-throughput manner. As discussed above, mitochondrial toxicities are classified into several categories (i.e., effects on ROS production, MMP depolarization) that are likely intertwined. Therefore, HTS for mitochondrial liabilities of drugs provides a means of accurate classification of such toxicities, which may prove critical to safety pharmacology. HTS techniques for mitochondrial liability detection usually include a self-defined combination of the following assays: HCS for mitochondrial content, arrangement, shape, MMP, and so on; microplate reader-based assays for ATP and ROS detection; and Seahorse assay for OCR measurement. HTS for mitochondrial liabilities is widely applied in a variety of cell types [354,365,368,383,384,385]. However, the use of HTS to detect cardiac mitochondrial toxicity is still in its infancy. Multiparametric analyses were performed by HCS to show the effects of the drugs on mitochondria in hiPSC-CMs [81]. An antibody against translocase of outer mitochondrial membrane 20 (TOM20) indicated mitochondrial changes similar to sarcomeres and nuclei induced by aspirin, doxorubicin, erlotinib, and sorafenib. Most notably, mitochondrial structure changes were detected at lower concentrations compared to the loss of contractility and cell count [386]. Furthermore, concentration–effect profiles of mitochondria-related changes correlated well with cell viability induced by cardiotoxic drugs [387]. Twenty-three cardiotoxicants were identified in 69 environmental hazards based on MMP evaluation by JC-10 staining in hiPSC-CMs [388]. Mitochondrial respiration analyzed by Seahorse assay was found to be a very sensitive and robust means of detecting mitotoxicity in hiPSC-CMs, and thus can be used both as a screening method and validation tool [307].

Several technical details are worth paying attention to when planning an HTS for mitotoxicity. Drug concentration and incubation time are critical for the identification of mitochondrial toxicity, and distinguishing them from cytotoxicity, in HTS assays. However, the existing literature does not fully distinguish between mitotoxicity and cytotoxicity, as evidenced by the frequent use of mitochondrial parameters as a surrogate of cellular conditions. Therefore, no consensus has yet been reached concerning the threshold separating these two entities. For example, 80% of drugs with hepatocyte toxicity were identified at a concentration of 100 μM or 30-fold of C_max_ with 3 days of incubation. TMRM staining indicated that 70% of those with cytotoxicity exhibited mitochondrial toxicity [383]. In cardiotoxicity drugs, the percentage of mitochondrial toxic drugs increased with increasing concentrations of drugs, ranging from 1 to 100 fold of C_max_, as evaluated by the glucose/galactose model in rat liver mitochondria [64], an insensitive mitotoxicity measurement [345]. Exposure of up to 100 fold of C_max_ over a period of 72 h was found to be essential for cytotoxicity examination of slower-acting toxicants in HCS [389]. On the contrary, the combined use of four metabolic biomarkers of toxicity (three of which were pertinent to mitochondria) achieved 90% sensitivity and 79% specificity in an assay using 10 fold of C_max_ in hiPSC-CMs [309]. Therefore, a concentration lower than that used to induce cytotoxicity would be useful for identifying compounds with primary actions on mitochondria. A shorter incubation time of 1 to 6 h [357,390] or 24 h (if requiring metabolism for mitochondrial toxicity) [359] also helps to distinguish mitochondrial toxicity from cytotoxicity. It is noteworthy that when the concentration of drug needed to induce mitochondrial toxicity is not significantly lower than that needed to induce cytotoxicity (IC_50_ ratio ≤ 3), it is difficult to determine whether mitochondrial toxicity is a primary or secondary effect of drug action, and further validation is therefore required to dissect the underlying mechanisms [390]. More detailed information on drug metabolism is also worthy of attention. Drugs, especially prodrugs, may be metabolized into active forms in cells, which do not easily diffuse back into the extracellular matrix and are thus accumulated in the cytoplasm, inducing a higher drug concentration than C_max_ [391]. This type of toxicity, including both mitochondrial toxicity and cytotoxicity, may not be discovered by exposing cells to the C_max_ concentration. 

### 4.4. Proposed Integrated Assays for Drug-Induced Mitochondria Toxicity of Cardiomyocytes

**Figure 3:**
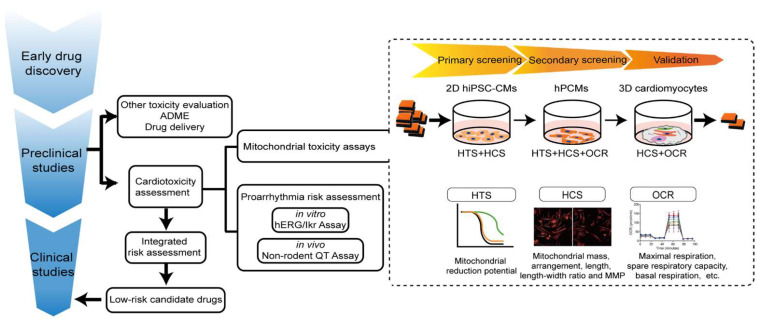
Proposed workflow of mitochondrial toxicity evaluation during preclinical cardiotoxicity profiling. Mitochondrial toxicity assays can be conducted in parallel with the existing proarrhythmic risk assessments to aid the selection of safer drugs for subsequent clinical studies. In our proposed workflow, 2D hiPSC-CMs, hPCMs, and 3D hiPSC-derived cardiomyocytes models can be cultured in 96- or 384-well assay plates, and treated with candidate drugs for primary screening, secondary screening, and subsequent validation, respectively. Primary assays can be performed firstly by PrestoBlue staining for measurement of reduction potential. Then, the fluorescent images can be captured in a high-content manner after MitoTracker and TMRM staining. Secondary screening combines microplate reading, HCS, and OCR measurement. Validation can be conducted with HCS and OCR measurement. These readouts (reduction potential, mitochondrial mass, distribution, morphology, MMP, and OCR) can be subsequently quantified to identify mitochondrially safe drugs. Abbreviations: ADME: absorption, distribution, metabolism, and excretion; hERG: human ether-a-go-go-related current; 2D hiPSC-CMs: two-dimensional human induced pluripotent-stem-cell-derived cardiomyocytes; HCS: high-content screening; HTS: high-throughput screening; OCR: oxygen consumption rate.

The prevalence of drug-induced mitochondrial cardiotoxicity warrants a more rigorous, systematic, and comprehensive evaluation of compounds early in the drug discovery process. HTS is a commonly used method for drug screening, and thus can also be utilized for the detection of mitochondrial liabilities of drugs. Since arrhythmia and mitochondrial dysfunction exist as two distinct entities in cardiotoxicity, we suggest an independent screening module that can be performed in parallel for proarrhythmic risk assessments to enhance predictive capabilities for cardiotoxicity (Figure 3). The choice of cellular model is pivotal to HTS. As discussed above, 2D and 3D hiPSC-derived cardiomyocytes and hPCMs models each have their advantages and drawbacks. On one hand, 2D hiPSC-CMs have been widely used due to their ease of scaled production, but do not sufficiently resemble the in vivo condition; on the other, hPCMs, while of native origin, can face practical problems, including tissue availability, isolation quality, and compatibility with HTS. According to our unpublished data, approximately three million hPCMs can be isolated from one milligram of heart tissue, and they can be further cultured and cryopreserved without morphological and functional alterations, indicating their compatibility with HTS. While 3D cardiomyocyte model assemblies are structurally and functionally advanced, however, their uniformity and scalability still need optimization.

An HTS approach for drug-induced mitochondrial toxicity can incorporate many of the aforementioned parameters. For example, primary screening can be performed by 2D hiPSC-CMs combining microplate reading and HCS of a variety of readouts, including mitochondrial reduction potential, mass, arrangement, length, length-to-width ratio, and MMP. In particular, cardiomyocytes cultured in 96- or 384-well assay plates can be firstly tested by PrestoBlue for reduction potential, then detected by HCS for the rest of the readouts (MitoTracker for mitochondrial mass and morphology, TMRM for MMP). Secondary screening and subsequent validation can be conducted in the hPCM and 3D cardiomyocyte models, respectively, by combining microplate reading, HCS, and OCR measurement. A mitochondrial toxicity index can be calculated as a weighted average of these readouts and can guide ranking of the cardiomyocyte-specific mitochondrial toxicity of compounds, and when combined with data from proarrhythmic risk assessment, can provide evidence for decisions regarding further development. 

Even if mitochondrial toxicity does not reach the level of discontinuation of drug development, the resultant data will provide an early warning sign of potential adverse reactions in a clinical setting, and may indicate measures for monitoring potential adverse events, such as lipoatrophy and peripheral neuropathy [392], and inform patient care. The combined preclinical cardiotoxicity assessments may also be useful for dissecting mechanisms of toxicity, such as the relationship between mitochondrial toxicity and excitation-contraction coupling or arrhythmias. Another benefit of screening for mitochondrial toxicity early in the drug discovery process is the early identification of structure–toxicity relationships to minimize or circumvent this liability from a chemical perspective. The recently identified 1,3-nitrogen motif in anticancer drugs was shown to inhibit MRC complex I in cardiomyocytes [140].

## 5. Conclusions and Future Perspectives

Mitochondria in cardiomyocytes ensure the proper functioning of the heart by producing energy and regulating redox balance, calcium homeostasis, and cell death [393,394]. Due to their mass and their central roles in cellular function, mitochondria in cardiomyocytes are particularly vulnerable to mitochondrial toxicants [22]. Cancer therapies, antiviral compounds, antibiotics, antidiabetic drugs, nonsteroidal anti-inflammatory agents, local anesthetics, and many other therapeutics often impair mitochondrial function [22]. Mitochondrial dysfunction is known to cause a broad spectrum of CVDs, including cardiomyopathies, arrhythmias, and abnormalities of the conduction system [395]. Therefore, cardiomyopathy, arrhythmias, and heart failure are the most common presentations of mitochondrial cardiotoxicity [36]. Up to 26% of patients treated with DOX exhibit cardiotoxicity with symptoms of cardiomyopathy [396], arrhythmia [397], and heart failure [398]. In addition, existing CVDs can also be aggravated by mitochondrion-toxic agents [36]. Despite the prevalence of mitochondrial toxicity and its impact on the heart, current clinical assessments of cardiac function are not able to detect subclinical myocardial dysfunction, let alone the underlying pathophysiology (e.g., mitochondrial toxicity) [399]. Nuclear imaging-based strategies with mitochondrial-potential- and ROS-targeted tracers for mitotoxicity in vivo have not yet achieved the desired sensitivity and molecular specificity for clinical assessments, but have the potential for future translation [400]. Here, we proposed a preclinical screening model for drug-induced mitochondrial toxicity of cardiomyocytes in HTS format, which can be performed in parallel with current proarrhythmic risk assessments for cardiac safety. Although this proposed workflow potentially improves and perfects the cardiac safety screening system, it is not intended to provide solid evidence of human cardiac toxicity, or lack thereof, in areas that exceed the scope of such screening (e.g., chronic toxicity).

While the mechanisms of drug toxicity are heavily studied in animal hearts, the real effect and mechanisms in human cardiomyocytes are less well understood [36], which prompted the development of HTS for mitochondrial toxicants in human-relevant platforms. In addition to drug-induced mitochondrial toxicity, accumulating studies have pointed out that environmental toxins, including various pesticides and heavy metals, may also induce cardiotoxicity [401,402]. Hence, HTS may be useful in applications beyond the regular drug discovery pipeline. In addition to cardiac safety assessment, HTS can be utilized to search for cardioprotective drugs and provide clues to their pharmacological actions. By applying mitochondrial toxicants with distinct mechanisms of action, the screen is capable of identifying different categories of cardioprotectancts. On the other hand, unexpected hits from such screens may be indicative of previously unknown drug actions. In a similar vein, since mitochondrial dysfunction is a common feature of many CVDs, HTS is a viable approach to finding mitotherapeutics for disease treatment, such as cardiomyopathy [403,404]. Screens intended to determine the mitochondrial liability of drugs may also reveal inter-relations of different toxicity phenotypes. For example, mitochondrial impairment by cardiotoxins was found to be an underlying cause of structural cardiotoxicity in hESC-CMs and H9c2 cells [288]. Given that Ca^2+^ handling, ATP production, and ROS signaling in mitochondria have all been shown to play important roles in arrhythmia, such as atrial fibrillation [401], it might be worth deciphering the relationship between drug-induced mitochondrial dysfunction and drug-induced arrhythmia. Furthermore, it is crucial to determine whether mitochondrial toxicants affect non-cardiovascular organ systems or the heart, particularly cardiomyocytes. For example, sertraline caused hepatotoxicity by uncoupling OXPHOS and inhibiting MRC complexes I and V [405]. Whether they also exert the same effects in cardiomyocytes is unclear. Screening for potential cardiac mitochondrial toxicity will contribute to identifying hidden cardiotoxicity and guiding clinical medication. 

## Figures and Tables

**Table 2:** Drugs affecting mitochondrial morphology, structure, MQC, their clinical manifestations, and relevant in vitro and in vivo studies.

Modules	Alterations	Pharmacology	Drugs	Clinical Manifestations	C_max_	Models	Dose	Time	References
Morphology	Mitochondrial swelling	Anthracyclines	DOX	CHF, decreased LVEF, ST, myocarditis, CMP	15.3 μM	Male Wistar rats (IP)	2&2.5 mg/kg/2 d	2 w	[94]
						Male Wistar rats (IP)	2.5 mg/kg/2 d	2 w	[95]
						Male Wistar rats (IV)	1 mg/kg/w	7 w (started at 11 w, observed at 48 w)	[96]
			Idarubicin	CMP, MI, CHF, VA, decreased LVEF	23.22 μM	Male SD rats (IV)	5 mg/kg/w	6 w	[110]
Morphology	Mitochondrial swelling	Alkylating agent	Cyclophosphamide	HMC, CMP	143 μM	Male Wistar rats (IP)	200 mg/kg	10 d	[111]
Morphology	Mitochondrial swelling	Chemotherapeutic agents	Cisplatin	Decreased LVEF, arrhythmias, ECA, myocarditis, CMP	27.54 μM	C57BL mice (IV)	10 mg/kg/d	1 w	[112]
Morphology	Mitochondrial swelling	Monoclonal antibody	Trastuzumab	CMP, LVD, CHF	2.59 mM	Female white New Zealand rabbits (SC)	8 mg/kg, a single dose; 8 mg/kg first w, 6 mg/kg for three additional w	4 w	[113]
Morphology	Mitochondrial swelling	TKIs	Sunitinib	Decreased LVEF, QT prolongation, TdP, hypertension, HF, CMP	0.25 μM	Patient			[114]
						Male SD rats (oral)	10 mg/kg/d	3 w	[89]
Morphology	Mitochondrial swelling	NSAIDs	Diclofenac	Hypertension, arrhythmias	7.9 µM	Isolated rat heart mitochondria	10 µg/mL	1 h	[115]
						Isolated rat heart mitochondria	50 μM	1 h	[60]
			Naproxen	-	100 µM	Isolated rat heart mitochondria	25 μM	1 h	[60]
			Celecoxib	Thrombosis, MI, stroke	3–5 µM	Isolated rat heart mitochondria	100 μM	1 h	[60]
Morphology	Mitochondrial swelling	NRTIs	Zidovudine	CMP	4 μM	Rats (oral)	125 mg/kg/d	4 w	[116]
Morphology	Mitochondrial swelling	Cardiac glycosides	*Nerium oleander* L.	PVB, AVB, VT	-	Guinea pigs (oral)	150&300 mg/kg	3 h	[117]
Morphology	Mitochondrial swelling	β-adrenoceptor agonists	Isoproterenol	HF	0.01 μM	Male Wistar rats (SC)	100 mg/kg, BID	12 h	[118]
						Male Wistar rats (SC)	100 mg/kg, BID	12 h	[83]
						Male Wistar rats (SC)	100 mg/kg, BID	12 h	[82]
			Propranolol	Cardiotoxicity	0.22 μM	Isolated rat heart mitochondria	5 µg/mL	5 min	[119]
			Atenolol	Cardiotoxicity	4.99 μM	Isolated rat heart mitochondria	10 µg/mL	5 min	[119]
Morphology	Mitochondrial swelling	Macrolide antibiotics	Azithromycin	Arrhythmia	0.32–0.87 μM	Isolated rat heart mitochondria	25 μM	1 h	[120]
			Clarithromycin	TdP	2.67–13.37 μM	Isolated rat heart mitochondria	50 μM	1 h	[120]
			Erythromycin	TdP	11 μM	Isolated rat heart mitochondria	25 μM	1 h	[120]
Morphology	Mitochondrial swelling	Aconitum species	Aconitum sp.	VA	19.27 μg/ml	H9c2	1 μM		[121]
Morphology	Mitochondrial swelling	Diabetes medication	Pioglitazone	HF	2.6 μM	Isolated rat heart mitochondria	12.5 µg/mL (30 min), 25 µg/mL (5 min)		[122]
Morphology	Morphological damage	NRTIs	Zidovudine	CMP	4 μM	H9c2	50 μM	39 passages	[123]
			Didanosine	CMP	12 μM	H9c2	50 μM	10 passages	[123]
Structure	Cristae disappearance	Chemotherapeutic agents	As_2_O_3_	QT prolongation TdP, CMP, tachycardia	12.1 μM	Male BALB/c mice	2 mg/kg	14 d	[84]
Structure	Cristae disappearance	Anthracyclines	DOX	CHF, decreased LVEF, ST, myocarditis, CMP	15.3 μM	Male Wistar rats (IP)	2&2.5 mg/kg/2 d	2 w	[94]
						Male Wistar rats (IP)	2.5 mg/kg/2 d	2 w	[95]
						Kunming mice (IP)	2 mg/kg	10 d	[102]
						Male Wistar rats (IV)	1 mg/kg/w	7 w (started at 11 w, observed at 48 w)	[96]
Structure	Cristae disappearance	Alkylating agent	Cyclophosphamide	HMC, CMP	143 μM	Male Wistar rats (IP)	200 mg/kg	10 d	[111]
						Male Wistar rats (IP)	200 mg/kg	10 d	[124]
						Patient			[125]
Structure	Cristae disappearance	TKIs	Sorafenib	Bleeding, hypertension,QT prolongation, CHF, CI, MI	16.6 μM	Male SD rats (oral)	10 mg/kg/d	3 w	[89]
Structure	Cristae disappearance	NRTIs	Zidovudine	CMP	4 μM	Rats (oral)	125 mg/kg/d	4 w	[116]
						Pregnant CD-1 mice + pups,oral	75 mg/kg, BID	2 w prior to pregnancy to pups postnatal 28 d	[126]
Structure	Cristae disappearance	β-adrenoceptor agonists	Isoproterenol	HF	0.01 μM	Male Wistar rats (SC)	100 mg/kg, BID	12 h	[118]
						Male Wistar rats (SC)	100 mg/kg, BID	12 h	[83]
						Male Wistar rats (SC)	100 mg/kg, BID	12 h	[82]
Structure	Cristae disorganization	Monoclonal antibody	Trastuzumab	CMP, LVD, CHF	2.59 mM	Female white New Zealand rabbits (SC)	8 mg/kg, a single dose; 8 mg/kg first W, 6 mg/kg for three additional w	4 w	[113]
Structure	OMM or/and IMM disruption	NRTIs	Zidovudine	CMP	4 μM	Rats (oral)	125 mg/kg/d	4 w	[116]
		Monoclonal antibody	Trastuzumab	CMP, LVD, CHF	2.59 mM	Female white New Zealand rabbits (SC)	8 mg/kg for first w, 6 mg/kg for three additional w	4 w	[113]
Structure	Matrix clearout	Anthracyclines	DOX	CHF, decreased LVEF, ST, myocarditis, CMP	15.3 μM	Male Wistar rats, intraperitoneal(IP)	2.5 mg/kg/2 d	2 w	[95]
Structure	Matrix clearout	TKIs	Sunitinib	Decreased LVEF, QT prolongation, TdP, hypertension, HF, CMP		Male SD rats (oral)	10 mg/kg/d	3 w	[89]
			Regorafenib	MI; hypertension		H9c2	10 μM	72 h	[90]
Structure		β-adrenoceptor agonists	Isoproterenol	HF	0.01 μM	Male Wistar rats (SC)	100 mg/kg, BID	12 h	[118]
	Matrix clearout					Male Wistar rats (SC)	100 mg/kg, BID	12 h	[83]
						Male Wistar rats (SC)	100 mg/kg, BID	12 h	[82]
Structure	Matrix clearout	Chemotherapeutic agents	Cisplatin	Decreased LVEF, arrhythmias, ECA, myocarditis, CMP	27.54 μM	C57BL mice (IV)	10 mg/kg/d	1 w	[112]
			As_2_O_3_	QT prolongation TdP, CMP, tachycardia	12.1 μM	Male BALB/c mice	2 mg/kg	14 d	[84]
MQC	Excessive mitophagy	Anthracyclines	DOX	CHF, decreased LVEF, ST, myocarditis, CMP QT prolongation TdP, CMP, tachycardia	15.3 μM	AC16 cells	15.625 nM	24 h	[127]
						Adult rat cardiac myocytes	1 μM	4 h	[128]
MQC	Excessive mitophagy	Chemotherapeutic agents	As_2_O_3_		12.1 μM	HL-1	6 μM	6 h	[129]
MQC	Inhibition of mitophagy	Aconitum species	Aconitum sp.	VA	19.27 μg/ml	H9c2	2 μM	24 h	[121]
MQC	Inhibition of mitochondrial biogenesis	Monoclonal antibody	Trastuzumab	CMP, LVD, CHF	2.59 mM	-	-	-	
MQC	Mitochondrial dynamics	TKIs	Sunitinib	Decreased LVEF, QT prolongation, TdP, hypertension, HF, CMP	0.25 μM	-	-	-	[130]
			Regorafenib	MI; hypertension	8.08 μM	H9c2	20 μM	48 h	[90]
MQC	Mitochondrial dynamics	NRTIs	Zidovudine	CMP	4 μM	Pregnant CD-1 mice + pups, oral	75 mg/kg, BID	2 w prior to pregnancy to pups postnatal 28 D	[126]
						TMPK-overexpressing H9c2 cells	100 µM	24 h	[131]
MQC	Mitochondrial dynamics	Nucleoside analogues	Remdesivir	Bradycardia, QT prologation, CA	9 μM	hiPSC-CMs	2.5 μM	3 d	[86]
MQC	Mitochondrial dynamics	Addictive drugs	Ethanol			H9c2	5 μM	0.5 h	[132]

Abbreviations: NRTIs: nucleoside reverse transcriptase inhibitors; NSAIDs: nonsteroidal anti-inflammatory drugs; TKIs: tyrosine kinase inhibitors; DOX: doxorubicin; CHF: congestive heart failure; LVEF: left ventricular ejection fraction; HF: heart failure; LVD: left ventricular dysfunction; TdP: torsades de pointes; MI: myocardial infarction; CMP: cardiomyopathy; CA: cardiac arrest; CI: cardiac ischemia; ST: sinus tachycardia; HMC: hemorrhagic myocarditis; ECA: electrocardiographic alterations; VA: ventricular arrhythmia; PVB: premature ventricular beats; AVB: atrioventricular block; VT: ventricular tachycardia; IP: intraperitoneal; IV: intravenously; SC: subcutaneously; BID: twice daily; w: week; d: day; h: hours.

**Table 3:** Drugs affecting OXPHOS, MMP and mPTP, their clinical manifestations, and relevant in vitro and in vivo studies.

Modules	Alterations	Pharmacology	Drugs	Clinical Manifestations	C_max_	Models	Dose	Time	References
OXPHOS	Inhibition of complex I	Cholesterol medications	Simvastatin	Cardiac atrophy	0.02 μM	H9c2	10 μM	24 h	[151]
OXPHOS	Inhibition of complex I	β-adrenoceptor agonists	Isoproterenol	HF	0.01 μM	Male Wistar rats (SC)	100 mg/kg, BID	12 h	[83]
OXPHOS	Inhibition of complex I	Alkylating agent	Cyclophosphamide	HMC, CMP	143 μM	Male Wistar rats (IP)	200 mg/kg	10 d	[111]
						Male Wistar rats (IP)	200 mg/kg	10 d	[124]
OXPHOS	Inhibition of complex I	NRTIs	Zidovudine	CMP	4 μM	Isolated mitochondria from H9c2	50 μM	3 passages	[152]
			Didanosine	CMP	12 μM	Isolated mitochondria from H9c2	50 μM	3 passages	[152]
OXPHOS	Inhibition of complex I	Anthracyclines	DOX	CHF, decreased LVEF, ST, myocarditis, CMP	15.3 μM	Male Wistar rats (IP)	2.5 mg/kg/2 d	2 w	[95]
OXPHOS	Inhibition of complex I	Chemotherapeutic agents	As_2_O_3_	QT prolongation TdP, CMP, tachycardia	12.1 μM	Isolated mitochondria from H9c2	5 μM	24 h	[141]
OXPHOS	Inhibition of complex I	Anesthesia	Propofol	HF, arrhythmia	30.13 μM	Cardiac muscle fibers of Wistar male rats	0.025 mM		[153]
			Halothane (fluothane)	-	10 μM	Pig heart submitochondrial particles	Dose response curve		[154]
	Inhibition of complex I	TKIs	Mubritinib	-	-	H9c2	0.5 μM		[140]
OXPHOS	Inhibition of complex I	NSAIDs	Nabumetone	-	2.45 μM	Submitochondrial particles	55 nmol/mg protein inhibit 50%		[142]
			Meclofenamate sodium	-	3.55 μM	Mitochontria	100 µM (70% inhibition)		[138]
			Naproxen	-	100 µM	Mitochontria	200 µM (50% inhibition)		[138]
OXPHOS	Inhibition of complex I	Addictive drugs	Cocaine	Arrhythmias, angina, MI, HF	0.76–0.94 µM	Isolated rat heart mitochondria	1 μM		[155]
OXPHOS	Inhibition of complex I	Anti-arrhythmic drug	Amiodarone	LQT, TdP, Hypotension, AV block, Arrhythmia, heart block, SBC, CHF, VF	4.65 μM	Isolated rat heart mitochondria	IC_50_ = 5.24 µM		[139]
			Dronedarone	AF, HF	0.15–0.26 μM	Isolated rat heart mitochondria	IC_50_ = 3.07 µM		[139]
OXPHOS	Inhibition of complex I	Immunosuppressant drug	Cyclosporine A	Cardiotoxicity	0.5–5 µM	Enzymes and coenzymes	100 µM		[156]
OXPHOS	Inhibition of complex II	NSAIDs	Diclofenac	Hypertension, arrhythmias	7.9 µM	Isolated rat heart mitochondria	10 µg/mL	1 h	[115]
			Naproxen	-	100 µM	Isolated rat heart mitochondria	50 μM	1 h	[60]
OXPHOS	Inhibition of complex II	Alkylating agent	Cyclophosphamide	HMC, CMP Cardiotoxicity	143 μM 0.22 μM	Male Wistar rats (IP)	200 mg/kg	10 d	[124]
						Male Wistar rats (IP)	200 mg/kg	10 d	[111]
OXPHOS	Inhibition of complex II	β receptor blocker drugs	Propranolol			Isolated rat heart mitochondria	10 µg/mL	30 min	[119]
			Atenolol	Cardiotoxicity	4.99 μM	Isolated rat heart mitochondria	10 µg/mL	30 min	[119]
OXPHOS	Inhibition of complex II	Macrolide antibiotics	Azithromycin	Arrhythmia	0.32–0.87 μM	Isolated rat heart mitochondria	25 μM	20 min	[120]
			Clarithromycin	TdP	2.67–13.37 μM	Isolated rat heart mitochondria	50 μM	20 min	[120]
			Erythromycin	TdP	11 μM	Isolated rat heart mitochondria	25 μM	20 min	[120]
OXPHOS	Inhibition of complex III	Chemotherapeutic agents	As_2_O_3_	QT prolongation TdP, CMP, tachycardia	12.1 μM	Isolated mitochondria from H9c2	5 μM	24 h	[141]
OXPHOS	Inhibition of complex III	TKIs	Sorafenib	Bleeding, hypertension, QT prolongation, CHF, CI, MI	16.6 μM	NRVMs	4.5 µM	20 min	[32]
OXPHOS	Inhibition of complex III	Alkylating agent	Cyclophosphamide	HMC, CMP	143 μM	Male Wistar rats (IP)	200 mg/kg	10 d	[111]
					7.9 µM	Male Wistar rats (IP)	200 mg/kg	10 d	[124]
OXPHOS	Inhibition of complex III	NSAIDs	Diclofenac	Hypertension, arrhythmias		Mitochondria isolated from mouse hearts	5 µM		[157]
			Meclofenamate sodium	-	3.55 μM	Mitochontria	10 µM		[138]
	Inhibition of complex III	Anthracyclines	DOX	CHF, decreased LVEF, ST, myocarditis, CMP	15.3 μM	-	15 mg/kg	-	[158]
OXPHOS	Inhibition of complex IV	Alkylating agent	Cyclophosphamide	HMC, CMP HF	143 μM 0.01 μM	Male Wistar rats (IP)	200 mg/kg	10 d	[111]
						Male Wistar rats (IP)	200 mg/kg	10 d	[124]
OXPHOS	Inhibition of complex IV	β-adrenoceptor agonists	Isoproterenol			Male Wistar rats (SC)	100 mg/kg, BID	12 h	[83]
OXPHOS	Inhibition of complex IV	Cholesterol medications	Simvastatin	Cardiac atrophy	0.02 μM	H9c2	10 μM	24 h	[151]
OXPHOS	Inhibition of complex IV	Anthracyclines	DOX	CHF, decreased LVEF, ST, myocarditis, CMP, QT prolongation TdP, CMP, tachycardia	15.3 μM 12.1 μM	Male Wistar rats (IP)	2.5 mg/kg/2 d	2 w	[95]
						Male Wistar rats (IV)	1 mg/kg/w	7 w(started at 11 w, observed at 48 w)	[96]
OXPHOS	Inhibition of complex IV	Chemotherapeutic agents	As_2_O_3_			Isolated mitochondria from H9c2	5 μM	24 h	[141]
OXPHOS	Inhibition of complex IV	NSAIDs	Celecoxib	Thrombosis, MI, stroke	3–5 µM	Isolated rat heart mitochondria	16 µg/mL		[14]
OXPHOS	Inhibition of complex IV	Proteasome inhibitor	Bortezomib	QT prolongation, hypotension	0.3 μM	Male Wistar rats	0.2 mg/kg	3 w	[159]
OXPHOS	Inhibition of complex IV	Immunosuppressant drug	Cyclosporine A	Cardiotoxicity	0.5–5 µM	Enzymes and coenzymes	100 µM		[156]
OXPHOS	Inhibition of complex V	Chemotherapeutic agents	Mitoxantrone	CHF, CMP, decreased LVEF, arrhythmia	3.3 μM	Isolated rat heart mitochondria	2.5 mg/kg on d 0, 10, and 20	22 d	[160]
OXPHOS	Inhibition of complex V	Anticonvulsants	Phenytoin	Bradycardia, hypotension	87.21 μM	guinea pig heart preparations	1.0 nM		[161]
OXPHOS	Downregulation of complex I expression	TKIs	Regorafenib	MI; hypertension	8.08 μM	H9c2	20 μM	72 h	[90]
OXPHOS	Downregulation of complex I expression	Nucleoside analogues	Remdesivir	Bradycardia, QT prologation, CA	9 μM	HiPSC-CMs	2.5 μM	3 d	[86]
OXPHOS	Downregulation of complex I expression	Addictive drugs	Ethanol			Male C57BL/6J mice	10% (*v*/*v*)	12 w	[162]
OXPHOS	Downregulation of complex I expression	Anthracyclines	DOX	CHF, decreased LVEF, ST, myocarditis, CMP	15.3 μM	Male CD-1 mice (IP)	9 mg/kg	1 w	[100]
			Mitoxantrone	CHF, CMP, decreased LVEF, arrhythmia	3.3 μM	Male CD-1 mice (IP)	6 mg/kg	1 w	[100]
OXPHOS	Downregulation of complexe II expression	Anesthesia	Propofol	HF, arrhythmia	30.13 μM	HiPSC-CMs	10 µg/mL	48 h	[163]
		Addictive drugs	Ethanol			Male C57BL/6J mice	10% (*v*/*v*)	12 w	[162]
OXPHOS	Downregulation of complex III expression	Addictive drugs	Ethanol			Male C57BL/6J mice	10% (*v*/*v*)	12 w	[162]
		Anthracyclines	DOX	CHF, decreased LVEF, ST, myocarditis, CMP	15.3 μM	Male CD-1 mice (IP)	9 mg/kg	1 w	[100]
OXPHOS	Downregulation of complex IV expression		Mitoxantrone	CHF, CMP, decreased LVEF, arrhythmia	3.3 μM	Male CD-1 mice (IP)	6 mg/kg	1 w	[100]
		Anthracyclines	DOX	CHF, decreased LVEF, ST, myocarditis, CMP	15.3 μM	Male Wistar rats (IV)	1 mg/kg/w	7 w(started at 11 w, observed at 48 w)	[96]
		Addictive drugs	Ethanol			Male C57BL/6J mice	10% (*v*/*v*)	12 w	[162]
OXPHOS	Downregulation of complex V expression	Nucleoside analogues	Remdesivir	Bradycardia, QT prologation, CA	9 μM	HiPSC-CMs	2.5 μM	3 d	[86]
OXPHOS	Downregulation of complex V expression	TKIs	Regorafenib	MI; hypertension	8.08 μM	H9c2	20 μM	72 h	[90]
OXPHOS	Downregulation of complex V expression	Proteasome inhibitor	Bortezomib	QT prolongation, hypotension	0.3 μM	Male Wistar rats	0.2 mg/kg	1 w	[159]
OXPHOS	Downregulation of complex V expression	Anthracyclines	DOX	CHF, decreased LVEF, ST, myocarditis, CMP	15.3 μM	Male CD-1 mice (IP)	9 mg/kg	1 w	[100]
			Mitoxantrone	CHF, CMP, decreased LVEF, arrhythmia	3.3 μM	Male CD-1 mice (IP)	6 mg/kg	1 w	[100]
OXPHOS	Downregulation of complex V expression	Nucleoside analogues	Remdesivir	Bradycardia, QT prologation, CA	9 μM	HiPSC-CMs	2.5 μM	3 d	[86]
OXPHOS	Downregulation of complex V expression	Addictive drugs	Ethanol			Male C57BL/6J mice	10% (*v*/*v*)	12 w	[162]
OXPHOS	Inhibition of OXPHOS	Anti-arrhythmic drug	Clofilium	TDP	1 μM	-	-	-	[64]
OXPHOS	Inhibition of OXPHOS	Antipsychotics	Aripiprazole	-	2.24 μM	-	-	-	[64]
OXPHOS	Inhibition of OXPHOS	TKIs	Sorafenib	Bleeding, hypertension, QT prolongation, CHF, CI, MI	16.6 μM	HiPSC-CMs	10 µM	24 h	[164]
OXPHOS	OCR reduction	NSAIDs	Acetylsalicylate	-	0.5–10 mM	Isolated rat heart mitochondria	5 mM		[165]
OXPHOS	OCR reduction	NRTIs	Zidovudine	CMP	4 μM	H9c2	50 μM	3 passages	[152]
						TMPK-overexpressing H9c2 cells	100 µM	24 h	[131]
			Didanosine	CMP	12 μM	H9c2	50 μM	3 passages	[152]
OXPHOS	OCR reduction	Nucleoside analogues	Remdesivir	Bradycardia, QT prologation, CA	9 μM	HiPSC-CMs	2.5 μM	3 d	[86]
OXPHOS	OCR reduction	Cholesterol medications	Simvastatin	Cardiac atrophy	0.02 μM	H9c2	10 μM	24 h	[151]
OXPHOS	OCR reduction	Analgesics	Salicylic acid	-	0.5–10 mM	Isolated rat heart mitochondria	5 mM		[165]
OXPHOS	OCR reduction	Local anesthetics	Bupivacaine (marcaine)	VF	0.7 μM	neonatal mouse cardiomyocytes	5 μM		[166]
OXPHOS	Reduction in ATP content	Anesthesia	Propofol	HF, arrhythmia	30.13 μM	Isolated rat heart mitochondria	300 μM		[167]
OXPHOS	Reduction in ATP content	Local anesthetics	Lidocaine	VF	36 μM	-	-	-	[168]
OXPHOS	Reduction in ATP content	Anthracyclines	DOX	CHF, decreased LVEF, ST, myocarditis, CMP	15.3 μM	-	15 mg/kg	-	[158]
OXPHOS	Reduction in ATP content	Chemotherapeutic agents	Etoposide	Hypotension	17 μM	hiPSC-CMs	30 μM	48 h	[169]
OXPHOS			Mitoxantrone	CHF, CMP, decreased LVEF, arrhythmia	3.3 μM				[160]
OXPHOS	Reduction in ATP content	Alkylating agent	Cyclophosphamide	HMC, CMP CMP, LVD, CHF	143 μM	Male Wistar rats (IP)	200 mg/kg	1 w	[170]
						Male Wistar rats (IP)	200 mg/kg	1 w	[171]
OXPHOS	Reduction in ATP content	Monoclonal antibody	Trastuzumab		2.59 mM	-	-	-	[172]
OXPHOS	Reduction in ATP content	TKIs	Imatinib mesylate	QT prolongation, CHF, decreased LVEF	2.71 μM	NRVMs	5 μM	24 h	[91]
			Sunitinib	Decreased LVEF, QT prolongation, TdP, hypertension, HF, CMP Bleeding, hypertension, QT prolongation, CHF, CI, MI	0.25 μM	Male SD rats (oral)	10 mg/kg/d	3 w	[89]
						Male Wistar Rats (oral)	25 mg/kg/d	28 d	[173]
						NRVMs	60% of ATP was depleted at 23 µM	24 h	[174]
			Sorafenib		16.6 μM	Male SD rats (oral)	10 mg/kg/d	3 w	[89]
			Regorafenib	MI; hypertension	8.08 μM	H9c2	5 μM	48 h	[90]
OXPHOS	Reduction in ATP content	NSAIDs	Naproxen	-	100 µM	Isolated rat heart mitochondria	50 μM	1 h	[60]
			Celecoxib	Thrombosis, MI, stroke	3–5 µM	Isolated rat heart mitochondria	25 μM	1 h	[60]
			Diclofenac	Hypertension, arrhythmias -	7.9 µM	Isolated rat heart mitochondria	100 μM	1 h	[60]
						-	-	-	[142]
						-	-	-	[175]
			Piroxicam		5 µM	-	-	-	[142]
			Indomethacin	Hypertension	6 µM	-	-	-	[142]
			Nimesulide	-	21.08 µM	-	-	-	[142]
			Meloxicam	HA, stroke	6.55 µM	-	-	-	[142]
OXPHOS	Reduction in ATP content	NRTIs	Zidovudine	CMP Bradycardia, QT prologation, CA	4 μM	Rats (oral)	125 mg/kg/d	4 w	[116]
						TMPK-overexpressing H9c2 cells	Dose response curve(IC_50_ = 70 μM)	4 d	[131]
OXPHOS	Reduction in ATP content	Nucleoside analogues	Remdesivir		9 μM				[176]
OXPHOS	Reduction in ATP content	Addictive drugs	Ethanol	Arrhythmias, angina, MI, HF		Male C57BL/6J mice	10% (*v*/*v*) for first w, 14% (*v*/*v*) for second w, 18% (*v*/*v*) for third w,	12 w	[162]
						H9c2	184.34 mM	24 h	[177]
			Cocaine	LQT, TdP, Hypotension, AV block, Arrhythmia, heart block, SBC, CHF, VF	0.76–0.94 µM	H9c2	1.79 mM	24 h	[177]
						Isolated rat heart mitochondria	2*7.5 mg/kg/d	7 d	[178]
						Isolated rat heart mitochondria	2*7.5 mg/kg/d	7 d	[179]
OXPHOS	Reduction in ATP content	Anti-arrhythmic drug	Amiodarone		4.65 μM	H9c2	IC_50_ = 1.84 µM	4 h	[139]
			Dronedarone	AF, HF	0.15–0.26 μM	H9c2	IC_50_ = 0.49 µM	4 h	[139]
OXPHOS	Reduction in ATP content	β-adrenoceptor agonists	Isoproterenol	HF	0.01 μM	Male Wistar rats (SC)	100 mg/kg, BID	12 h	[82]
OXPHOS	Reduction in ATP content	Cholesterol medications	Simvastatin	Cardiac atrophy	0.02 μM	H9c2	10 μM (6 h); 100 μM (24 h)		[151]
OXPHOS	Uncoupling of OXPHOS	TKIs	Crizotinib	QT prolongation	0.73 μM	-	-	-	[64]
OXPHOS	Uncoupling of OXPHOS	NSAIDs	Acetylsalicylate	-	0.5–10 mM	Isolated rat heart mitochondria	10 mM		[165]
			Diclofenac	Hypertension, arrhythmias	7.9 µM	-	-	-	[142]
			Piroxicam	-	5 µM	-	-	-	[142]
			Indomethacin	Hypertension	6 µM	-	-	-	[142]
			Nimesulide	-	21.08 µM	-	-	-	[142]
			Meloxicam	HA, stroke	6.55 µM	-	-	-	[142]
			tenidap	-	8.44 µM (30525499)	-	-	-	[64]
OXPHOS	Uncoupling of OXPHOS	NRTIs	Zidovudine	CMP	4 μM	H9c2	50 lM	18 h	[180]
			Didanosine	CMP	12 μM		50 lM	18 h	[180]
OXPHOS	Uncoupling of OXPHOS	Addictive drugs	Ethanol			Isolated mitochondria from rabbit ventricle	10 µM	2 h	[181]
OXPHOS	Uncoupling of OXPHOS	Anti-arrhythmic drug	Amiodarone	LQT, TdP, Hypotension, AV block, Arrhythmia, heart block, SBC, CHF, VF	4.65 μM	Isolated rat heart mitochondria	1 µM		[139]
			Dronedarone	AF, HF	0.15–0.26 μM	Isolated rat heart mitochondria	0.1 µM		[139]
OXPHOS	Uncoupling of OXPHOS	Analgesics	Salicylic acid	-	0.5–10 mM	Isolated rat heart mitochondria	10 mM		[165]
MMP	Dissipation of MMP	Anthracyclines	DOX	CMP, MI, CHF, VA, pericarditis, myocarditis	15.3 μM	Kunming mice (IP)	2 mg/kg	10 d	[102]
						KIND-2-derived cardiac cells	0.24 μM disrupte 48.3%	48 h	[182]
			Daunorubicin		89 μM	Neonatal rat cardiac cells	4 μM	24 h	[97]
MMP	Dissipation of MMP	Chemotherapeutic agents	Cisplatin	Decreased LVEF, arrhythmias, ECA, myocarditis, CMP Hypotension	27.54 μM	C57BL mice (IV)	10 mg/kg/d	1 W	[112]
						NRVMs	200 μM	24 h	[183]
			Etoposide		17 μM	HiPSC-CMs	10 μM	48 h	[169]
			As_2_O_3_	QT prolongation TdP, CMP, tachycardia	12.1 μM	H9c2	5 μM	24 h	[184]
MMP	Dissipation of MMP	Monoclonal antibody	Trastuzumab	CMP, LVD, CHF	2.59 mM	H9c2	200 nM	24 h	[185]
MMP	Dissipation of MMP	TKIs	Imatinib mesylate	QT prolongation, CHF, decreased LVEF	2.71 μM	NRVMs	5 μM	18 h	[91]
			Sunitinib	Decreased LVEF, QT prolongation, TdP, hypertension, HF, CMP	0.25 μM	Male SD rats (oral)	10 mg/kg/d	3 W	[89]
			Regorafenib	MI; hypertension	8.08 μM	H9c2	20 μM	72 h	[90]
MMP	Dissipation of MMP	NSAIDs	Diclofenac	Hypertension, arrhythmias -	7.9 µM		-	-	[142]
							Isolated rat heart mitochondria	10 µg/mL	[115]
							Mitochondria isolated from mouse hearts	10 µg/mL	[157]
							C57BL/6 mice (oral)	15 mg/kg/D	[175]
							Immortalized human cardiomyocytes	100 μM	[85]
			Piroxicam		5 µM		-	-	[142]
			Indomethacin	Hypertension	6 µM		-	-	[142]
			Nimesulide	-	21.08 µM		-	-	[142]
			Meloxicam	HA, stroke	6.55 µM		-	-	[142]
			Meclofenamate sodium	-	3.55 μM		H9c2	5 µM (40% inhibition)	[138]
			Naproxen	-	100 µM	Isolated rat heart mitochondria	25 μM (60 min); 100 μM (30 min)		[60]
			Diclofenac			Isolated rat heart mitochondria	50 μM	5 min	[60]
			Celecoxib	Thrombosis, MI, stroke	3–5 µM	Isolated rat heart mitochondria	25 μM	5 min	[60]
MMP	Dissipation of MMP	NRTIs	Zidovudine	CMP	4 μM	TMPK-overexpressing H9c2 cells	100 µM	24 h	[131]
MMP	Dissipation of MMP	Anti-arrhythmic drug	Amiodarone	LQT, TdP, Hypotension, AV block, Arrhythmia, heart block, SBC, CHF, VF	4.65 μM	H9c2	IC_50_ = 2.94 μM	6 h	[139]
			Dronedarone	AF, HF	0.15–0.26 μM	H9c2	IC_50_ = 0.5 μM	6 h	[139]
MMP	Dissipation of MMP	β receptor blocker drugs	Propranolol	Cardiotoxicity	0.22 μM	Isolated rat heart mitochondria	5 µg/mL	5 min	[119]
			Atenolol	Cardiotoxicity	4.99 μM	Isolated rat heart mitochondria	5 µg/mL	5 min	[119]
MMP	Dissipation of MMP	Aconitum species	Aconitum sp.	VA	19.27 μg/ml	H9c2	10 μM	24 h	[186]
MMP	Dissipation of MMP	Cholesterol medications	Simvastatin	Cardiac atrophy	0.02 μM	H9c2	10 μM	24 h	[151]
MMP	Dissipation of MMP	Diabetes medication	Pioglitazone	HF	2.6 μM	Isolated rat heart mitochondria	12.5 µg/mL	5 min	[122]
MMP	Dissipation of MMP	Anesthesia	Propofol	HF, arrhythmia	30.13 μM	Isolated rat heart mitochondria	300 μM		[167]
MMP	Dissipation of MMP	β-adrenoceptor agonists	Isoproterenol	HF	0.01 μM	Isolated rat heart mitochondria	85 mg/kg/d	2 d	[187]
mPTP	Increases in mPTP opening	NRTIs	Zidovudine	CMP	4 μM	TMPK-overexpressing H9c2 cells	100 µM	24 h	[131]
mPTP	Increases in mPTP opening	Chemotherapeutic agents	As_2_O_3_	QT prolongation TdP, CMP, tachycardia	12.1 μM	Male BALB/c mice	2 mg/kg (14 d); 4 mg/kg (3 d)		[84]
mPTP	Increases in mPTP opening	Monoclonal antibody	Trastuzumab	CMP, LVD, CHF	2.59 mM	-	-	-	[22]
mPTP	Loss of cytochrome c	Anthracyclines	DOX	CHF, decreased LVEF, ST, myocarditis, CMP	15.3 μM	Isolated rat heart mitochondria, subcutaneously (SC)	2 mg/kg/w	7 w	[188]
mPTP	Loss of cytochrome c	Chemotherapeutic agents	Cisplatin	Decreased LVEF, arrhythmias, ECA, myocarditis, CMP	27.54 μM	NRVMs	200 μM	24 h	[183]
mPTP	Loss of cytochrome c	TKIs	Imatinib mesylate	QT prolongation, CHF, decreased LVEF	2.71 μM	NRVMs	5 μM	24 h	[91]
mPTP	Loss of cytochrome c	NRTIs	Zidovudine	CMP	4 μM	Rats (oral)	125 mg/kg/d		[116]
mPTP	Loss of cytochrome c	β receptor blocker drugs	Propranolol	Cardiotoxicity	0.22 μM	Isolated rat heart mitochondria	5 µg/mL	5 min	[119]
			Atenolol	Cardiotoxicity	4.99 μM	Isolated rat heart mitochondria	10 µg/mL	5 min	[119]
mPTP	Loss of cytochrome c	Macrolide antibiotics	Azithromycin	Arrhythmia	0.32–0.87 μM	Isolated rat heart mitochondria	50 μM		[120]
			Clarithromycin	TdP	2.67–13.37 μM	Isolated rat heart mitochondria	50 μM		[120]
			Erythromycin	TdP	11 μM	Isolated rat heart mitochondria	50 μM		[120]
mPTP	Loss of cytochrome c	Diabetes medication	Pioglitazone	HF	2.6 μM	Isolated rat heart mitochondria	12.5 µg/mL		[122]

Abbreviations: OXPHOS: oxidative phosphorylation; MMP: mitochondrial membrane potential; mPTP: mitochondrial permeability transition pore; NRTIs: nucleoside reverse transcriptase inhibitors; NSAIDs: nonsteroidal anti-inflammatory drugs; TKIs: tyrosine kinase inhibitors; DOX: doxorubicin; LVEF: left ventricular ejection fraction; HF: heart failure; LVD: left ventricular dysfunction; TdP: torsades de pointes; CHF: congestive hearts failure; MI: myocardial infarction; AF: atrial fibrillation; CMP: cardiomyopathy; VF: ventricular fibrillation; CA: cardiac arrest; CI: cardiac ischemia; ST: sinus tachycardia; HMC: hemorrhagic myocarditis; SBC: sinus bradycardia; ECA: electrocardiographic alterations; VA: ventricular arrhythmia; HA: heart attack; IP: intraperitoneal; IV: intravenously; SC: subcutaneously; BID: twice daily; w: week; d: day; h: hours.

**Table 4:** Drugs affecting FA oxidation and TCA cycle, their clinical manifestations, and relevant in vitro and in vivo studies.

Modules	Alterations	Pharmacology	Drugs	Clinical Manifestations	C_max_	Models	Dose	Time	References
FA oxidation	Downregulation of FA oxidation related proteins expression	Anthracyclines	DOX	CHF, decreased LVEF, ST, myocarditis, CMP	15.3 μM	Male CD-1 mice (IP)	9 mg/kg	1 w	[100]
			Mitoxantrone	CHF, CMP, decreased LVEF, arrhythmia	3.3 μM	Male CD-1 mice (IP)	6 mg/kg	1 w	[100]
FA oxidation	Downregulation of FA oxidation related proteins expression	Alkylating agent	Cyclophosphamide	HMC, CMP	143 μM	Male Wistar rats (IP)	200 mg/kg	10 d	[189]
TCA cycle	Downregulation of TCA related proteins expression	Anthracyclines	DOX	CHF, decreased LVEF, ST, myocarditis, CMP	15.3 μM	Male CD-1 mice (IP)	9 mg/kg	1 w	[100]
			Mitoxantrone	CHF, CMP, decreased LVEF, arrhythmia	3.3 μM	Male CD-1 mice (IP)	6 mg/kg	1 w	[100]
TCA cycle	Inhibition of the Krebs cycle enzyme	NSAIDs	Acetylsalicylate	-	0.5–10 mM	Isolated rat heart mitochondria	Dose response curve		[165]
TCA cycle	Inhibition of the Krebs cycle enzyme	Analgesics	Salicylic acid	-	0.5–10 mM	Isolated rat heart mitochondria	Dose response curve		[165]
TCA cycle	Inhibition of the Krebs cycle enzyme	Anthracyclines	DOX	CHF, decreased LVEF, ST, myocarditis, CMP	15.3 μM	Male Wistar rats (IP)	2.5 mg/kg/2 d	2 w	[95]
TCA cycle	Inhibition of the Krebs cycle enzyme	Alkylating agent	Cyclophosphamide	HMC, CMP	143 μM	Male Wistar rats (IP)	200 mg/kg	10 d	[124]
						Male Wistar rats (IP)	200 mg/kg	10 d	[111]
TCA cycle	Inhibition of the Krebs cycle enzyme	β-adrenoceptor agonists	Isoproterenol	HF	0.01 μM	Male Wistar rats (SC)	100 mg/kg, BID	12 h	[83]
TCA cycle	Loss of Krebs cycle enzymes	Addictive drugs	Ethanol			Wistar male albino rats	3 g/kg/d	10 d	[190]

Abbreviations: FA: fatty acid; TCA: tricarboxylic acid; NRTIs: nucleoside reverse transcriptase inhibitors; NSAIDs: nonsteroidal anti-inflammatory drugs; DOX: doxorubicin; LVEF: left ventricular ejection fraction; HF: heart failure; CHF: congestive hearts failure; CMP: cardiomyopathy; ST: sinus tachycardia; HMC: hemorrhagic myocarditis; IP: intraperitoneal; SC: subcutaneously; BID: twice daily; w: week; d: day; h: hour.

**Table 5:** Drugs affecting mitochondrial redox, their clinical manifestations, and relevant in vitro and in vivo studies.

Modules	Alterations	Pharmacology	Drugs	Clinical Manifestations	C_max_	Models	Dose	Time	References
Redox	Decrease in antioxidant enzyme level	NSAIDs	Naproxen	-	100 µM	Isolated rat heart mitochondria	25 μM		[60]
			Celecoxib	Thrombosis, MI, stroke	3–5 µM	Isolated rat heart mitochondria	50 μM		[60]
			Diclofenac	-	3.55 μM	Isolated rat heart mitochondria	25 μM		[60]
Redox	Decrease in antioxidant enzyme level	β-adrenoceptor agonists	Isoproterenol	HF	0.01 μM	Male Wistar rats (SC)	100 mg/kg, BID	12 h	[118]
Redox	Inhibition of antioxidant enzyme	Anthracyclines	DOX	CHF, decreased LVEF, ST, myocarditis, CMP	15.3 μM	Male Wistar rats (IP)	2.5 mg/kg/2 d	2 w	[95]
						Kunming mice (IP)	2 mg/kg	10 d	[102]
						-	-	-	[199]
						Male BALB/c mice (IP)	5 mg/kg/w	2 w	[200]
						Male Wistar rats (IV)	45 mg/kg	48 h	[203]
			Idarubicin	CMP, MI, CHF, VA, decreased LVEF	23.22 μM	Rats (IV)	5 mg/kg/w	6 w	[110]
Redox	Inhibition of antioxidant enzyme	Alkylating agent	Cyclophosphamide	HMC, CMP	143 μM	Male Wistar rats	200 mg/kg	1 w	[170]
						Male Wistar rats (IP)	200 mg/kg	1 w	[171]
Redox	Inhibition of antioxidant enzyme	Chemotherapeutic agents	Cisplatin	Decreased LVEF, arrhythmias, ECA, myocarditis, CMP	27.54 μM	NRVMs	200 μM	24 h	[183]
			As_2_O_3_	QT prolongation TdP, CMP, tachycardia	12.1 μM	BALB/c mice (IV)	1 mg/kg/2 d	6 d	[204]
						Isolated mitochondria from H9c2	5 μM	24 h	[141]
Redox	Inhibition of antioxidant enzyme	TKIs	Sunitinib	Decreased LVEF, QT prolongation, TdP, hypertension, HF, CMP	0.25 μM	NRVMs	67% of GSH was oxidized at 23 µM	24 h	[174]
Redox	Inhibition of antioxidant enzyme	NRTIs	Zidovudine	CMP	4 μM	Male OF1 mice (oral)	10 mg/kg/d	35 d	[205]
Redox	Inhibition of antioxidant enzyme	Addictive drugs	Cocaine	Arrhythmias, angina, MI, HF	0.76–0.94 µM	H9c2	1.79 mM	24 h	[177]
Redox	Inhibition of antioxidant enzyme	β-adrenoceptor agonists	Isoproterenol	HF	0.01 μM	Male Wistar rats (SC)	100 mg/kg, BID	12 h	[83]
Redox	ROS elevation	Anthracyclines	DOX	CHF, decreased LVEF, ST, myocarditis, CMP	15.3 μM	Beef heart submitochondrial preparations	-	-	[206]
						-	-	-	[199]
			Daunorubicin	CMP, MI, CHF, VA, pericarditis, myocarditis	89 μM	-	-	-	[207]
			Idarubicin	CMP, MI, CHF, VA, decreased LVEF	23.22 μM	-	-	-	[207]
Redox	ROS elevation	Chemotherapeutic agents	Cisplatin	Decreased LVEF, arrhythmias, ECA, myocarditis, CMP	27.54 μM	NRVMs	200 μM	24 h	[183]
			Etoposide	Hypotension	17 μM	HiPSC-CMs	10 μM	48 h	[169]
			As_2_O_3_	QT prolongation TdP, CMP, tachycardia	12.1 μM	Male BALB/c mice	2 mg/kg (14 d); 4 mg/kg (7 d)		[84]
						Isolated mitochondria from H9c2	5 μM	24 h	[141]
						H9c2	5 μM	24 h	[184]
Redox	ROS elevation	Monoclonal antibody	Trastuzumab	CMP, LVD, CHF	2.59 mM	H9c2	200 nM	24 h	[185]
Redox	ROS elevation	TKIs	Sorafenib	Bleeding, hypertension, QT prolongation, CHF, CI, MI	16.6 μM	NRVMs	4.5 µM	10 min	[32]
Redox	ROS elevation	NSAIDs	Diclofenac	Hypertension, arrhythmias	3.55 μM 7.9 µM	Isolated rat heart mitochondria	25 μM	5 min	[60]
						H9c2	10 µM	1.5 h	[157]
						Isolated rat heart mitochondria	10 µg/mL	1 h	[115]
						C57BL/6 mice (oral)	15 mg/kg/d	4 w	[175]
						Immortalized human cardiomyocytes	100 μM	24 h	[85]
			Naproxen	-	100 µM	Isolated rat heart mitochondria	25 μM	5 min	[60]
			Celecoxib	Thrombosis, MI, stroke	3–5 µM	Isolated rat heart mitochondria	25 μM	5 min	[60]
Redox	ROS elevation	NRTIs	Zidovudine	CMP	4 μM	H9c2	50 μM	3 passages	[152]
						TMPK-overexpressing H9c2 cells	100 µM	24 h	[131]
						Human cardiomyocytes	10 µM	48 h	[198]
			Didanosine	CMP	12 μM	H9c2	50 μM	3 passages	[152]
Redox	ROS elevation	Addictive drugs	Ethanol			H9c2	184.34 mM	24 h	[177]
			Cocaine	Arrhythmias, angina, MI, HF	0.76–0.94 µM	H9c2	1.79 mM	24 h	[177]
						Isolated rat heart mitochondria	2 × 7.5 mg/kg/d	8 d	[178]
						Isolated rat heart mitochondria	2 × 7.5 mg/kg/d	7 d	[179]
Redox	ROS elevation	β-adrenoceptor agonists	Isoproterenol	HF	0.01 μM	Isolated rat heart mitochondria	85 mg/kg/d	2 d	[187]
Redox	ROS elevation	β receptor blocker drugs	Propranolol	Cardiotoxicity	0.22 μM	Isolated rat heart mitochondria	5 µg/mL	5 min	[119]
			Atenolol	Cardiotoxicity	4.99 μM	Isolated rat heart mitochondria	5 µg/mL	30 min	[119]
		Macrolide antibiotics	Azithromycin	Arrhythmia	0.32–0.87 μM	Isolated rat heart mitochondria	25 μM	15 min	[120]
			Clarithromycin	TdP	2.67–13.37 μM	Isolated rat heart mitochondria	25 μM	15 min	[120]
			Erythromycin	TdP	11 μM	Isolated rat heart mitochondria	25 μM	15 min	[120]
Redox	ROS elevation	Diabetes medication	Pioglitazone	HF	2.6 μM	Isolated rat heart mitochondria	12.5 µg/mL	5 min	[122]
Redox	ROS elevation	Local anesthetics	Bupivacaine (marcaine)	VF	0.7 μM	H9c2	1 mM	24 h	[208]
Redox	Nitrozative stress	Anthracyclines	Epirubicin	CHF	5.68 mM	Male Wistar rats (IP)	10 mg/kg	10 d	[209]
Redox	Nitrozative stress	Alkylating agent	Cyclophosphamide	HMC, CMP	143 μM	Male Wistar rats (IP)	200 mg/kg	1 w	[171]
Redox	8OHdG adducts in mtDNA	Anthracyclines	DOX	CHF, decreased LVEF, ST, myocarditis, CMP	15.3 μM	SD rats (IP)	2 mg/kg/w	6 w	[210]
Redox	Lipid peroxidation	Anthracyclines	DOX	CHF, decreased LVEF, ST, myocarditis, CMP	15.3 μM	Male Wistar rats (IP)	2.5 mg/kg/2 d	2 w	[95]
						Male Wistar rats (IV)	45 mg/kg	48 h	[203]
			Daunorubicin	CMP, MI, CHF, VA, pericarditis, myocarditis	89 μM	Male SD rats	2.5 mg/kg/w	5 w	[211]
			Idarubicin	CMP, MI, CHF, VA, decreased LVEF	23.22 μM	Male SD rats (IV)	5 mg/kg/w	6 w	[110]
Redox	Lipid peroxidation	Alkylating agent	Cyclophosphamide	HMC, CMP	143 μM	Male Wistar rats	200 mg/kg	1 w	[170]
Redox	Lipid peroxidation	Chemotherapeutic agents	Cisplatin	Decreased LVEF, arrhythmias, ECA, myocarditis, CMP	27.54 μM	NRVMs	200 μM	24 h	[183]
Redox	Lipid peroxidation	NSAIDs	Diclofenac	Hypertension, arrhythmias	7.9 µM	Isolated rat heart mitochondria	50 μM	1 h	[60]
						Isolated rat heart mitochondria	10 µg/mL	1 h	[115]
			Naproxen	-	100 µM	Isolated rat heart mitochondria	100 μM	1 h	[60]
			Celecoxib	Thrombosis, MI, stroke	3–5 µM	Isolated rat heart mitochondria	100 μM	1 h	[60]
Redox	Lipid peroxidation	NRTIs	Zidovudine	CMP	4 μM	Male OF1 mice (oral)	10 mg/kg/d	35 d	[205]
Redox	Lipid peroxidation	β-adrenoceptor agonists	Isoproterenol	CHF, decreased LVEF, ST, myocarditis, CMP	0.01 μM	Rat, subcutaneously (SC)	100 mg/kg, BID	12 h	[118]
						Male Wistar rats (SC)	100 mg/kg, BID	12 h	[83]

Abbreviations: ROS: reactive oxygen species; NRTIs: nucleoside reverse transcriptase inhibitors; NSAIDs: nonsteroidal anti-inflammatory drugs; TKIs: tyrosine kinase inhibitors; DOX: doxorubicin; CHF: congestive heart failure; LVEF: left ventricular ejection fraction; HF: heart failure; LVD: left ventricular dysfunction; TdP: torsades de pointes; MI: myocardial infarction; CMP: cardiomyopathy; VF: ventricular fibrillation; CI: cardiac ischemia; ST: sinus tachycardia; HMC: hemorrhagic myocarditis; ECA: electrocardiographic alterations; VA: ventricular arrhythmia; HA: heart attack; IP: intraperitoneal; IV: intravenously; SC: subcutaneously; BID: twice daily; w: week; d: day; h: hours.

## Data Availability

Not applicable.
